# *SnRK2.10* in apple positively regulates the nitrogen use efficiency via modulating the nitrogen uptake

**DOI:** 10.1111/tpj.71139

**Published:** 2026-10-05

**Authors:** Ying Ye, Zisheng Zuo, Zhaoting Li, Lin Luo, Xiaojun Huang, Yuduan Ding, Fengwang Ma, Xiaoqing Gong

**Affiliations:** ^1^ State Key Laboratory of Crop Stress Biology for Arid Areas/Shaanxi Key Laboratory of Apple, College of Horticulture Northwest A&F University Yangling 712100 Shaanxi People's Republic of China

**Keywords:** apple, nitrogen stress, SnRK2.10, transport protein, NUE

## Abstract

Sucrose non‐fermentation‐1–related protein kinase 2 (SnRK2) family members play key roles in plant adaptation to environmental stress. In this study, low nitrogen (N) stress induced the expression of *SnRK2.10*, which mitigated the adverse effects of N deficiency in apple plants. *SnRK2.10*‐overexpressing (OE) plants showed enhanced growth and higher nitrogen use efficiency (NUE), whereas *SnRK2.10*‐silenced (Ri) plants exhibited more severe growth inhibition and reduced NUE, compared with the wild‐type (WT) plants under low N conditions. The transcriptomic and proteomic analyses indicated that *SnRK2.10* regulates N metabolism and transport. It physically interacted with the nitrate transporter NRT2.5 and the ammonium transporter AMT1.1. Consistently, OE plants displayed higher NO_3_^−^ and NH_4_^+^ uptake rates than WT and Ri plants under low N stress. Functional analyses using transgenic apple lines with different expression levels of *NRT2.5* or *AMT1.1* demonstrated that both transporters positively regulate growth and NUE in apple plants' response to low N stress. Co‐expression of *SnRK2.10* with either *NRT2.5* or *AMT1.1* effectively altered plant growth under low N stress. Collectively, these results indicate that *SnRK2.10* positively regulates plant growth and NUE under low N stress, probably by promoting N uptake through direct interaction with key N transporters.

## Significance Statement

Boosting nitrogen use efficiency in perennial fruit crops sustains yields and cuts fertilizer inputs and environmental harm. This study reveals that SnRK2.10 mediates apple adaptation to low‐nitrogen conditions via regulating nitrogen uptake, providing valuable genetic resources for breeding cultivars with high nitrogen use efficiency.

## INTRODUCTION

Nitrogen (N) is an essential nutrient for plant growth and development and a major component of amino acids, enzymes, and diverse metabolites (Perchlik & Tegeder, 2018). It regulates multiple aspects of plant metabolism, growth, and development, including seed germination (Cheng et al., 2021), root system establishment (Ma et al., 2014), organ development (Li et al., 2024), starch accumulation (Ullah et al., 2023), and secondary metabolism (Comadira et al., 2015). Adequate N supply enhances photosynthesis, crop yield, and root growth (Sadras & Richards, 2014), whereas N deficiency severely restricts plant growth and development (Huché‐Thélier et al., 2011). To adapt to low‐N conditions, plants have evolved coordinated morphological, physiological, and molecular responses. For example, in Arabidopsis, nitrate‐regulated root growth is mediated by ABA accumulation at the root tip to maintain meristem activity (Ondzighi‐Assoume et al., 2016). Under limited nitrate availability, ABA also improves nitrate uptake by regulating the expression of *NRT2/NAR* genes (Wang et al., 2020). In apple rootstocks, the transcription factor *bHLH130* modulates N uptake under low‐N stress by regulating flavonoid and lignin biosynthesis (Wang et al., 2023).

Sucrose non‐fermenting 1‐related protein kinases (SnRKs) are major protein kinases involved in plant signal transduction and are key components of cascade signaling pathways. They function as central regulators of cellular energy and metabolic balance (Boudsocq et al., 2004; Robaglia et al., 2012) and serve as hubs integrating nutrient metabolism, energy status, and stress‐response signaling (Coello et al., 2011). SnRK2s are core components of the ABA‐dependent signaling pathway and operate within the PYL‐PP2C‐SnRK2 module, which underpins plant responses to abiotic stress (Takahashi et al., 2020). Extensive evidence shows that *SnRK2s* participate in plant responses to salinity, drought, and cold stress. In *Arabidopsis thaliana*, *SnRK2.4* and *SnRK2.10* regulate reactive oxygen species (ROS) homeostasis under salt stress; *SnRK2.4* controls hydrogen peroxide levels, peroxidase activity, and ascorbic acid content, whereas *SnRK2.10* is rapidly activated by salt and enhances tolerance by sustaining photosynthesis and limiting oxidative damage (Mazur et al., 2021; Szymanska et al., 2019). In *Solanum nigrum*, *PtrSnRK2.4* enhances drought tolerance by promoting putrescine biosynthesis (Song et al., 2022). In rice, *OsSAPK8* is strongly induced by drought, and its mutant shows lower drought tolerance at the vegetative stage and decreases yield (Zhong et al., 2020). Under low‐temperature conditions, *OsSAPK8* mutants exhibit retarded growth, while overexpression of *TaSnRK2.3* in *Arabidopsis* or transfer of *AcSnRK2.11* into tobacco significantly improves cold tolerance (Tian et al., 2013; Xiang et al., 2020; Zhong et al., 2020).

Nitrogen uptake and transport in plants are mediated primarily by nitrate transporters (NRTs) and ammonium transporters (AMTs). The high‐affinity nitrate transporter NRT2 plays a key role in plant adaptation to low N conditions. *Arabidopsis* contains seven NRT2 members, five of which function in nitrate uptake across different tissues (Lv et al., 2022). Among them, NRT2.4 is localized to the plasma membrane of lateral root epidermal cells and stem tissues near the phloem, where it contributes to root nitrate uptake and long‐distance nitrate transport (Kiba et al., 2012). Under N deficiency, induction and plasma‐membrane localization of NRT2.5 are essential for adult plant growth and for efficient nitrate uptake and allocation to the phloem (Lezhneva et al., 2014). In addition, *NRT2.5* and *NRT2.6* jointly respond to rhizobium infection and counteract the suppression of lateral root development caused by high N availability (Dechorgnat et al., 2012). Dof1.7 regulates *NRT2* expression in an N‐dependent manner: under N‐sufficient conditions, it directly activates *NRT2.5*, whereas under N deficiency it indirectly activates *NRT2.1* and *NRT2.4* by repressing *NIGT1* expression (Zhuo et al., 2024). Following root nitrate uptake, NO_3_^−^ is reduced to NH_4_^+^ in plant cells. Ammonium transport is primarily mediated by AMT family proteins, and their expression is dynamically regulated by plant nitrogen status (Bajgain et al., 2018). For instance, exogenous nitrate supply strongly represses *AMT1.2* expression in multiple plant species (Gonçalves et al., 2020). Transcriptional regulation constitutes a major layer of control for *AMT* gene expression, involving a diverse array of transcription factors belonging to different families. For example, the conserved bZIP transcription factor HY5 negatively regulates *AtAMT1.2* in *Arabidopsis* and *OsAMT1.1* in rice, while the rice DOF‐type transcription factor OsDOF18 exerts positive regulatory effects on *OsAMT1.3*, *OsAMT2.1*, and *OsAMT4.1* (Huang et al., 2015; Wu et al., 2017).

Apple is an economically important fruit tree whose productivity is strongly affected by environmental stress. SnRKs are central regulators integrating nutrient metabolism and stress‐response signaling. Although SnRKs have been linked to N utilization (Lu et al., 2015), their role in apple responses to N deficiency remains unclear. We previously identified 12 *SnRK2s* in the apple genome and demonstrated that *SnRK2.10* enhances drought and salt tolerance (Shao et al., 2019; Ye et al., 2022). In this study, we investigated the role of *SnRK2.10* in low N stress responses and NUE in apple plants. We generated multiple transgenic apple lines targeting *SnRK2.10* and two N transporter genes, *NRT2.5* and *AMT1.1*. Our results show that *SnRK2.10* positively regulates plant growth and NUE under low N conditions. SnRK2.10 directly interacts with NRT2.5 and AMT1.1, which may contribute to the enhanced N uptake of the apple plants during N deficiency. Collectively, our findings identify a SnRK2.10‐NRT2.5/AMT1.1 regulatory module that enhances N acquisition under low N stress, providing a theoretical basis and genetic resources for improving NUE and stress tolerance in apple breeding and production.

## RESULTS

### SnRK2.10 positively regulates the low N response and NUE in apple plants

SnRK2.10 was localized in both the cytoplasm and nucleus (Figure 1B), and its expression was induced by low N stress. Expression increased nearly 4‐fold at 4 days of low N treatment in leaves and nearly 7.5‐fold at 5 days of low N treatment in roots (Figure 1A). In addition, strong *SnRK2.10* signals were detected in vascular tissues, including xylem and phloem, suggesting a potential role in N uptake in apple plants (Figure 1C). To verify the role of *SnRK2.10* in regulating low N stress responses and NUE in apple plants, we used two previously generated transgenic apple lines overexpressing *SnRK2.10* (OE1 and OE2; Figure 2; Shao et al., 2019). Under control conditions, no significant differences in growth phenotypes were observed between OE and WT plants. However, after 30 days of low N stress, the aerial parts of OE plants were similar to those of WT plants, whereas OE plants developed more extensive root systems than WT plants (Figure 2A). Under low N stress, OE plants exhibited higher Pn values than WT plants (Figure 2B), and both root dry weight and root activity (DHA) were also higher in OE plants (Figure 2C,D). Measurement of N content showed that OE plants had higher N content than WT plants (Figure 2E). Consequently, NUE was significantly higher in OE plants than in WT plants under low N stress (Figure 2F). To further confirm the role of *SnRK2.10* in low N stress responses, we generated transgenic apple plants with suppressed SnRK2.10 expression (Ri1, Ri2, and Ri3; Figure S1A,B). Under control conditions, Ri plants showed growth comparable to WT plants, while under low N stress, Ri plants were smaller than WT plants (Figure 2G). Moreover, all enhanced physiological traits observed in OE plants were reversed in Ri plants, including Pn, root dry weight, DHA, N content, and NUE, which were all lower in Ri plants than in WT plants (Figure 2H–L). Together, these results indicate that *SnRK2.10* acts as a positive regulator of low N stress responses and NUE in apple plants.

**Figure 1:**
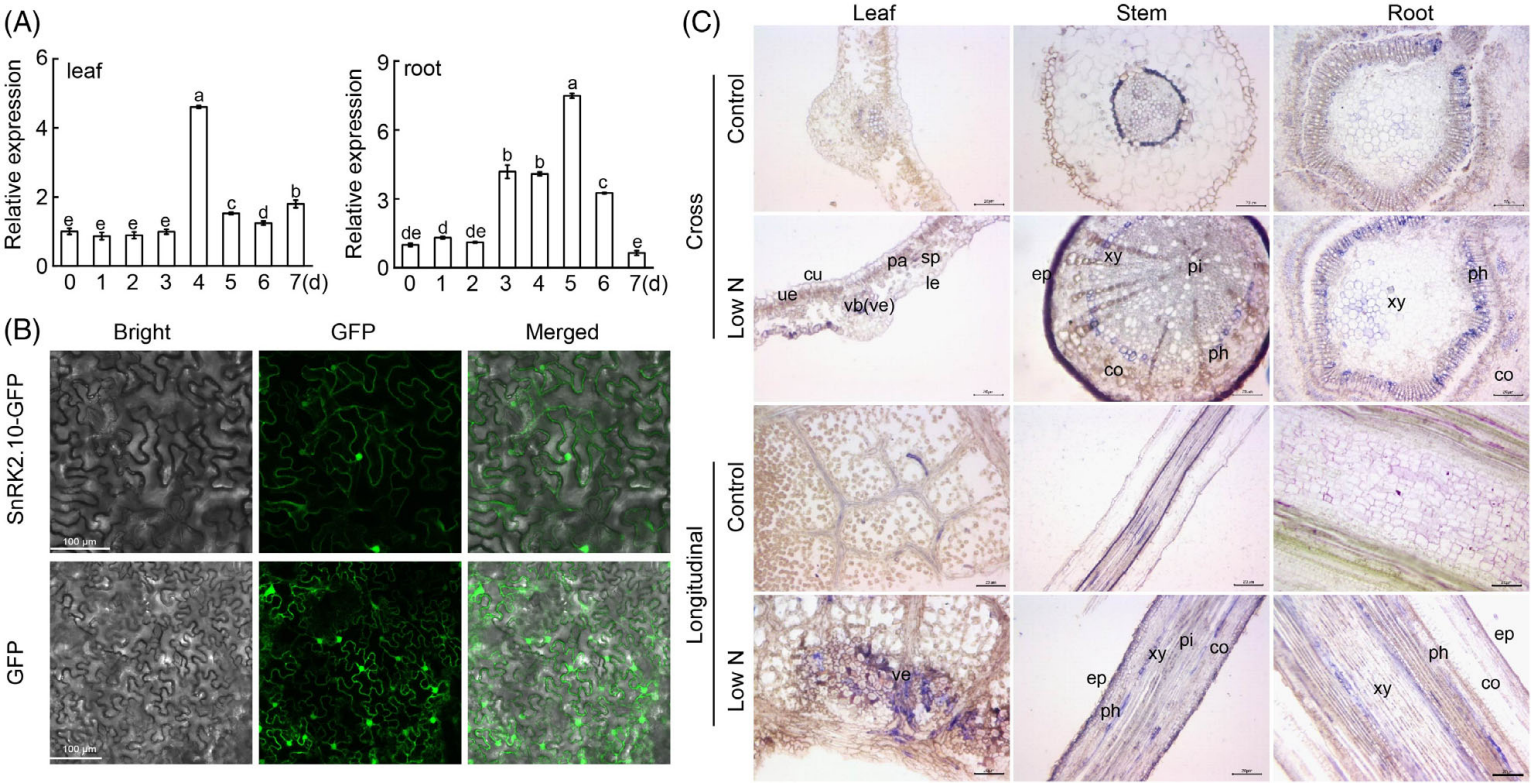
Expression patterns and subcellular localization of *SnRK2.10*. (A) The expression level of *SnRK2.10* in the leaves and roots under low N stress. (B) Subcellular localization of apple SnRK2.10. Scale bars = 100 μm; BF, bright field; GFP, green fluorescent protein. (C) *In situ* hybridization of SnRK2.10 in roots, leaves, and stems of GL‐3 apple (*Malus domestica*) under control conditions (CK) and under low N conditions (low N). ep, epidermis; xy, xylem; ph, phloem; pi, pith; co, cortex; cu, cuticle; ue, upper epidermis; le, lower epidermis; pa, palisade mesophyll cells; sp, spongy mesophyll cells; vb, vascular bundle; ve, vein. Scale bar = 20 μm. Data are mean ± SD. According to one‐way ANOVA and Duncan multiple range test, different letters represent significant differences between different plants (*P* < 0.05). CK, plants without any treatment; low N, low N stress treatment (0.02 mM N).

**Figure 2:**
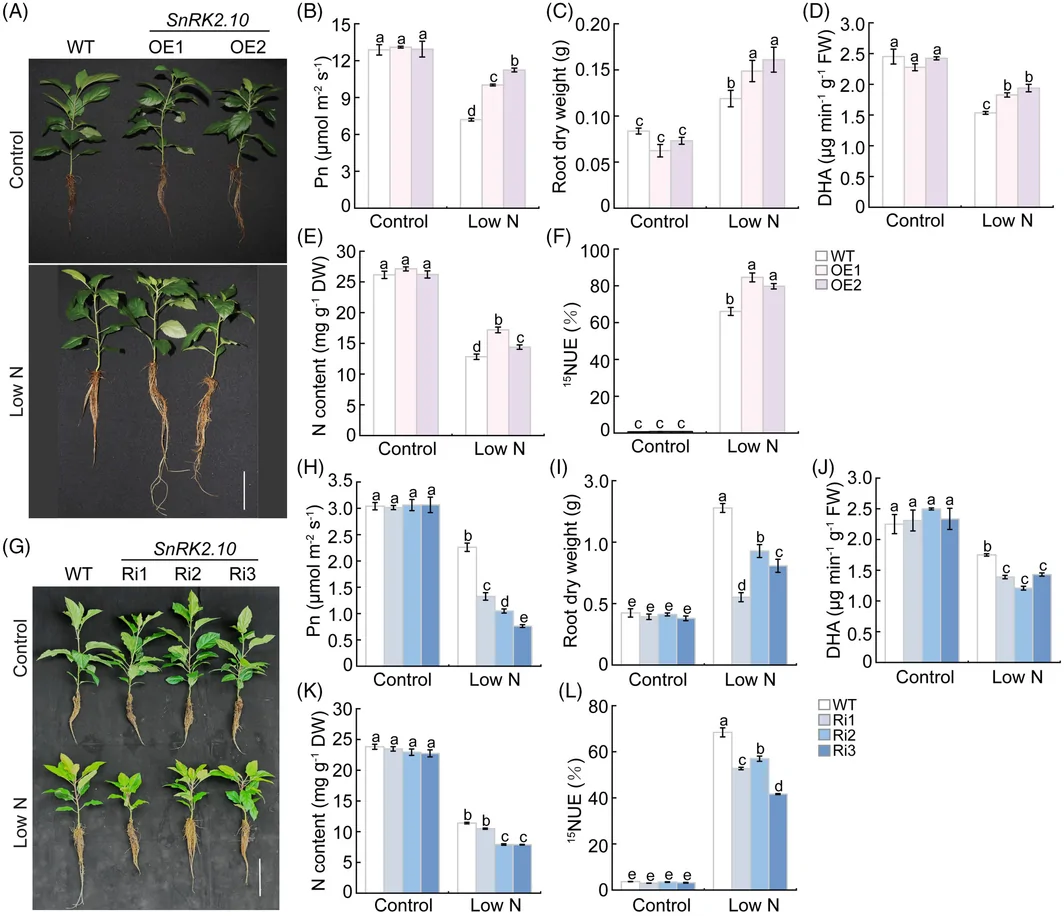
*SnRK2.10* overexpression enhanced the tolerance of apple plants to low‐N stress. (A) Phenotypes of WT and *SnRK2.10*‐OE apple lines after 30 days of cultivation under control or low N conditions. Scale bar: 10 cm. (B) Net photosynthesis (Pn) in WT and *SnRK2.10*‐OE apple lines. (C) Root dry weight in WT and *SnRK2.10*‐OE apple lines. (D) DHA in WT and *SnRK2.10*‐OE apple lines. (E) N content in WT and *SnRK2.10*‐OE apple lines. (F) NUE in WT and *SnRK2.10*‐OE apple lines. (G) Phenotypes of WT and *SnRK2.10*‐Ri apple lines after 30 days of cultivation under control or low N conditions. Scale bar = 10 cm. (H) Net photosynthesis (Pn) in WT and *SnRK2.10*‐Ri apple lines. (I) Root dry weight in WT and *SnRK2.10*‐Ri apple lines. (J) DHA in WT and *SnRK2.10*‐Ri apple lines. (K) N content in WT and *SnRK2.10*‐Ri apple lines. (L) NUE in WT and *SnRK2.10*‐Ri apple lines. Data are mean ± SD. According to one‐way ANOVA and Duncan multiple range test, different letters represent significant differences among all the collected data (*P* < 0.05). WT, wild type, here we used GL‐3 apple (*Malus domestic*), which was also used as explants in generating transgenic apple plants; OE1/2, *SnRK2.10*‐overexpression line 1/2; Ri1/2/3, *SnRK2.10*‐RNA interference line 1/2/3.

### Effects of SnRK2.10 on the transcriptional and translational levels in apple plants under low N stress

To investigate the biological basis by which *SnRK2.10* regulates the apple response to low N stress, RNA‐seq analysis was first performed using OE (OE2) and WT plants. Using a 1.5‐fold change threshold, 4966 DEGs were identified in the OE/WT comparison (Figure S2B), including 1761 up‐regulated and 3205 down‐regulated genes (Figure S2A). Clustering analysis showed that many DEGs were associated with N nutrition. Specifically, down‐regulated DEGs in the OE/WT group were mainly enriched in RNA regulation, cell wall organization, central amino acid metabolism, and N transport (Figure S2C, down group). In contrast, up‐regulated DEGs were mainly enriched in RNA regulation, hormone metabolism, N metabolism, and DNA synthesis (Figure S2C, up group). These results suggest that the major transcriptional differences between OE and WT plants occur in RNA regulation and N nutrition. In addition, proteomic analysis identified 857 DEPs in the OE/WT comparison (Figure S3B), including 281 up‐regulated and 576 down‐regulated proteins, using a 1.2‐fold threshold (Figure S3A). Clustering of DEPs showed that many were also associated with N nutrition, particularly N transport (Figure S3C). A total of 17 N‐transport‐related DEGs were identified in the RNA‐seq data, including 3 AMTs, 10 DEGs encoding NRTs, and 4 encoding nitrate antiporters, with most showing higher expression in OE plants than in WT (Figure 3A; Table S2). In contrast, only 6 N‐transport–related DEPs were detected, including 3 AMTs, 2 NRTs, and 1 nitrate antiporter (Figure 3B, Supplemental Table 2), all of which were upregulated in OE plants. Notably, *AMT1.1 (MD13G1225400)* and *NRT2.5 (MD13G1096700)* were identified in both the DEG and DEP datasets. We performed qPCR analysis to examine the expression levels of these two genes in *SnRK2.10*‐OE and WT plants. The results showed that both *AMT1.1* and *NRT2.5* were expressed much higher in OE lines than in WT, and this pattern was also maintained under low nitrogen stress (Figure 3C), consistent with the RNA‐seq data. These results suggested that NRT2.5 and AMT1.1 may be the direct targets of SnRK2.10.

**Figure 3:**
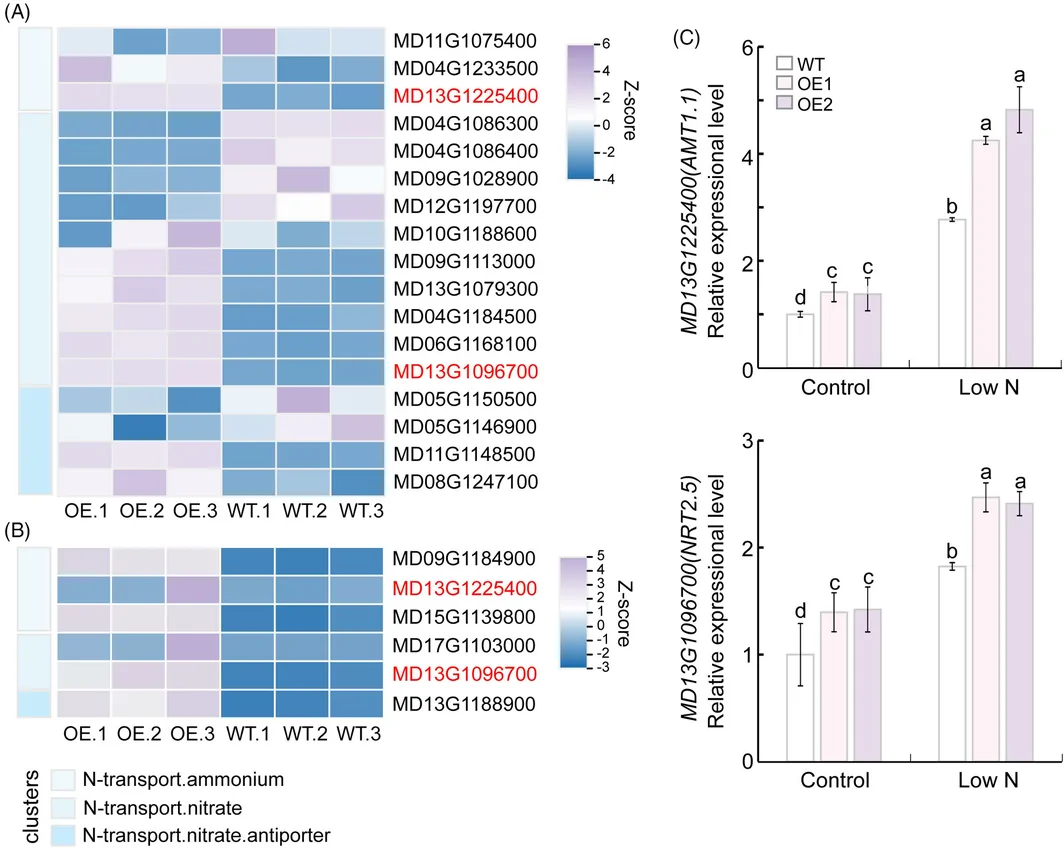
Through multi‐omics analysis, the nitrate transporter NRT2.5 and the ammonium transporter AMT1.1 were screened. (A) The relative quantitative expression levels of N metabolism‐related genes in the transcriptome. (B) The relative quantitative expression levels of N metabolism‐related proteins in the proteome. The colors indicate the expression of each DEG (A) and DEP (B) as determined by the average peak response area with *Z*‐score normalization. (C) Relative expression levels of *AMT1.1* and *NRT2.5* in WT and *SnRK2.10*‐overexpressing lines. Data are mean ± SD. According to one‐way ANOVA and Duncan multiple range test, different letters represent significant differences among all the collected data (*P* < 0.05). WT, wild type, here we used GL‐3 apple (*Malus domestica)*, which was also used as explants in generating transgenic apple plants; OE1/2 in (C), *SnRK2.10*‐overexpression line 1/2; WT.1/2/3 and OE.1/2/3 in (A) and (B) represent three biological replicates.

### SnRK2.10 interacts with the N transporters and promotes N uptake under low N stress

To further verify whether SnRK2.10 interacts with NRT2.5 and AMT1.1, Y2H assays were performed. Yeast cells co‐transformed with SnRK2.10 and NRT2.5 or AMT1.1 grew well on ‐Trp/−Leu/‐His/−Ade medium (Figure 4A). When SnRK2.10 and NRT2.5 or AMT1.1 were co‐expressed in tobacco leaves, strong luciferase signals were detected using an ultra‐sensitive imaging system (Figure 4B,C). In pull‐down assays, purified SnRK2.10‐GST was incubated with NRT2.5‐His or AMT1.1‐His, and protein interactions were confirmed after pull‐down (Figure 4D,E). Collectively, these results demonstrate that SnRK2.10 interacts with NRT2.5 and AMT1.1, suggesting that SnRK2.10 may promote NUE by enhancing N uptake in apple plants.

**Figure 4:**
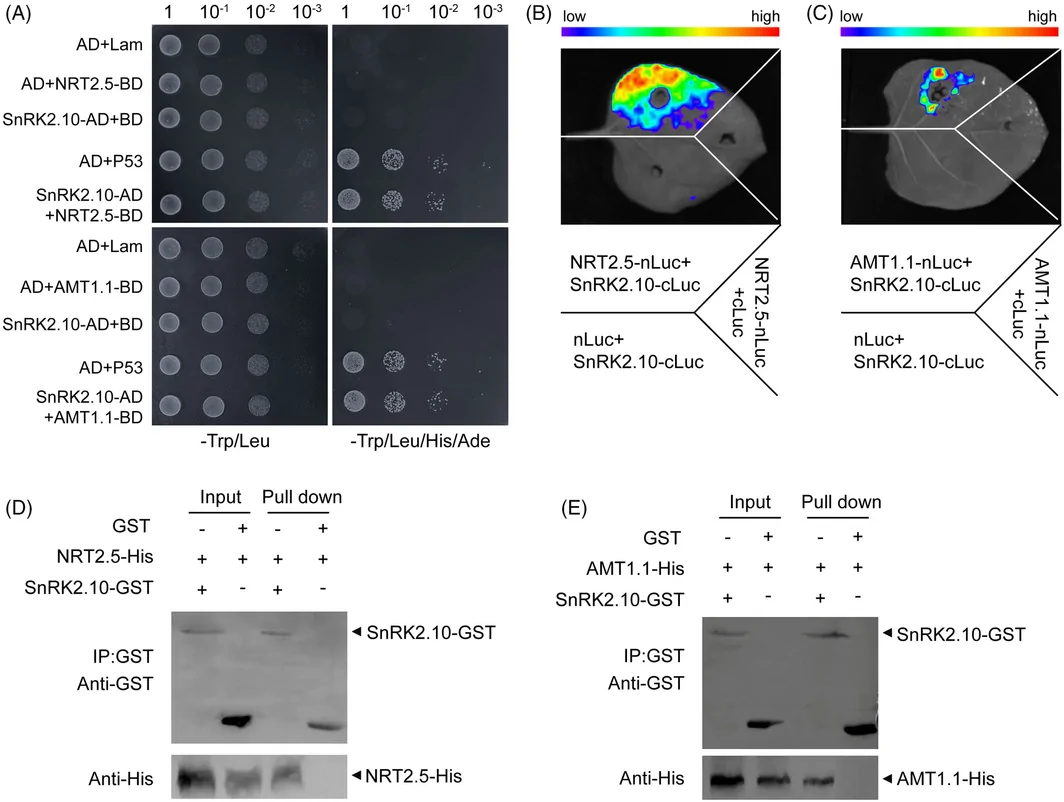
SnRK2.10 interacts directly with NRT2.5 and AMT1.1. (A) A Y2H assay indicates the interaction of SnRK2.10 and NRT2.5 or AMT1.1. (B) A Split‐LUC assay indicates the interaction of SnRK2.10 and NRT2.5. (C) A Split‐LUC assay indicates the interaction of SnRK2.10 and AMT1.1. (D) A pull‐down assay indicates the interaction of SnRK2.10 and NRT2.5. (E) A pull‐down assay indicates the interaction of SnRK2.10 and AMT1.1. Split‐LUC, split luciferase complementation assay; Y2H; yeast two‐hybrid.

We used NMT to measure the ion flow rates of NO_3_^−^ and NH_4_^+^ in the roots of WT and *SnRK2.10* transgenic apple plants. The results showed that under control conditions, there was no significant difference in nitrate ion flow rates among the different plant lines. Under low N stress, nitrate flow rates significantly decreased in all plants. The nitrate flow rate in the OE plants was significantly higher than that in the WT plants, whereas the nitrate flow rate in the Ri plants was significantly lower than that in the WT plants (Figure 5A,B). Furthermore, under control conditions, there was no significant difference in NH_4_^+^ excretion rates among the different lines. However, under low N treatment, NH_4_^+^ absorption increased in all plants. The NH_4_^+^ absorption rate in the OE lines was significantly higher than that in the WT plants, whereas the rate in the Ri lines was significantly lower than that in the WT plants (Figure 5C,D). Although the low N conditions repressed net NO_3_^−^ influx, its absolute values were markedly greater than net NH_4_^+^ influx, suggesting that SnRK2.10 appears to preferentially regulate NRT2.5 in apple. These results suggested that SnRK2.10 promotes NUE in apple plants under low N stress, probably by regulating N uptake.

**Figure 5:**
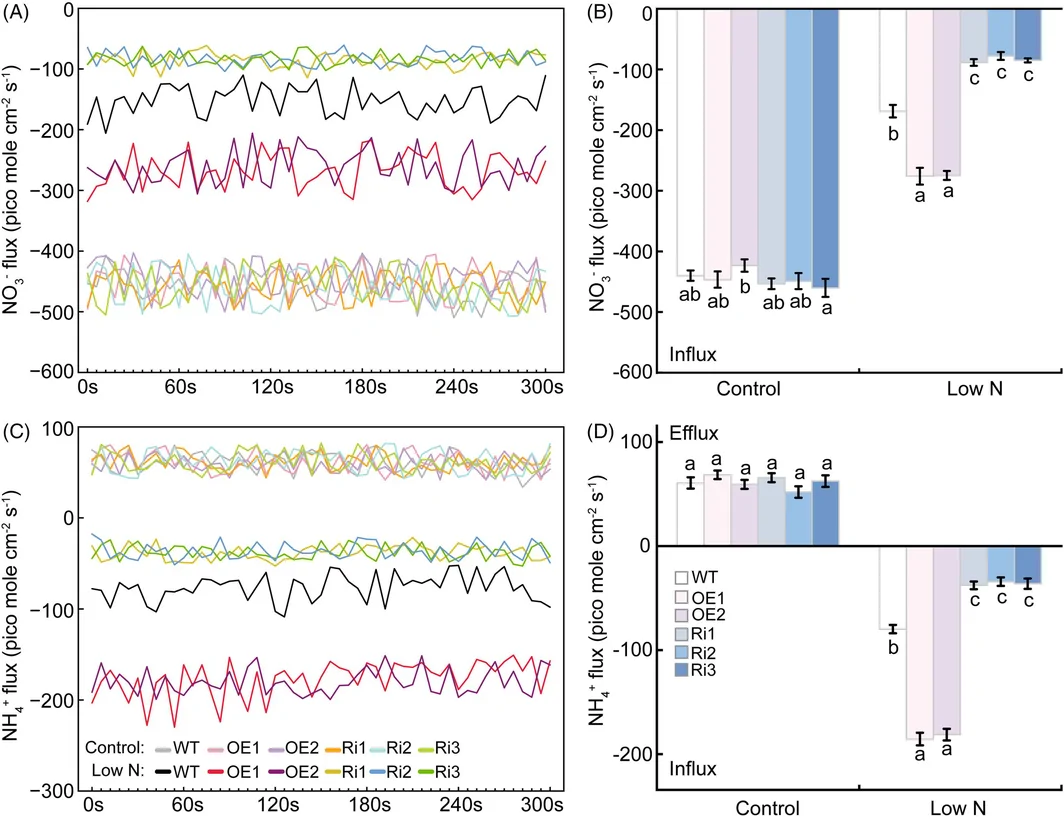
Effects of NO_3_^−^ flux and NH_4_^+^ flux in each line. (A, B) Effects of low N stress on net NO_3_^−^ flux in apple plants with different expression levels of *SnRK2.10*. (C, D) Effects of low N stress on net NH_4_^+^ flux in apple plants with different expression levels of *SnRK2.10*. The positive or negative sign of the flow rate value represents the direction of ion transport, with the positive as efflux and the negative as influx. Data are mean ± SD. According to one‐way ANOVA and Duncan multiple range test, different letters represent significant differences among different lines under control and low N stress, respectively (*P* < 0.05). WT, wild type, here we used GL‐3 apple (*Malus domestic*), which was also used as explants in generating transgenic apple plants; OE1/2, *SnRK2.10*‐overexpression line 1/2; Ri1/2/3, *SnRK2.10*‐RNA interference line 1/2/3.

### NRT2.5 positively regulates the NUE of apple plants under low N stress

The expression of *NRT2.5* in leaves increased 2.5‐fold after 4 days of low N treatment, while its expression in roots increased 5‐fold after 5 days of low N treatment (Figure S4A). *In situ* hybridization showed strong *NRT2.5* signals in vascular tissues, including the xylem and phloem (Figure S4C), similar to those observed for *SnRK2.10*. This protein was mainly localized in the cytoplasm (Figure S4B). To investigate the role of *NRT2.5* in the low N stress response of apple plants, we generated transgenic lines overexpressing *NRT2.5* (OE1, OE2, and OE3; Figure S1C,D) and lines with suppressed *NRT2.5* expression (Ri1, Ri2, and Ri3; Figure S1E,F). Under control conditions, no significant differences in growth phenotypes were observed between transgenic and WT plants. However, after 30 days of low N stress, OE plants showed milder leaf chlorosis, whereas Ri plants exhibited more severe leaf chlorosis compared with WT plants (Figure 6A,B). Although Pn decreased in all plants under low N stress, Pn values were significantly higher in OE lines than in WT plants and were lowest in Ri lines (Figure 6D). Under control conditions, chlorophyll fluorescence and Fv/Fm values did not differ between WT and transgenic plants. Under low N stress, the chlorophyll fluorescence was stronger in OE plants than in WT plants, and weakest in Ri plants (Figure 6C). Correspondingly, Fv/Fm values significantly decreased in all lines, but remained highest in OE plants, intermediate in WT plants, and lowest in Ri plants (Figure 6E). Additionally, OE plants developed stronger root systems, whereas Ri plants exhibited weaker root systems compared with WT plants (Figure 6A). Furthermore, under low N stress, root dry matter accumulation was higher in OE plants and lower in Ri plants relative to WT plants (Figure 6F). Root activity, assessed by DHA, decreased in all lines under low N stress. However, OE plants maintained the highest DHA values, Ri plants the lowest, and WT plants intermediate values (Figure 6G).

**Figure 6:**
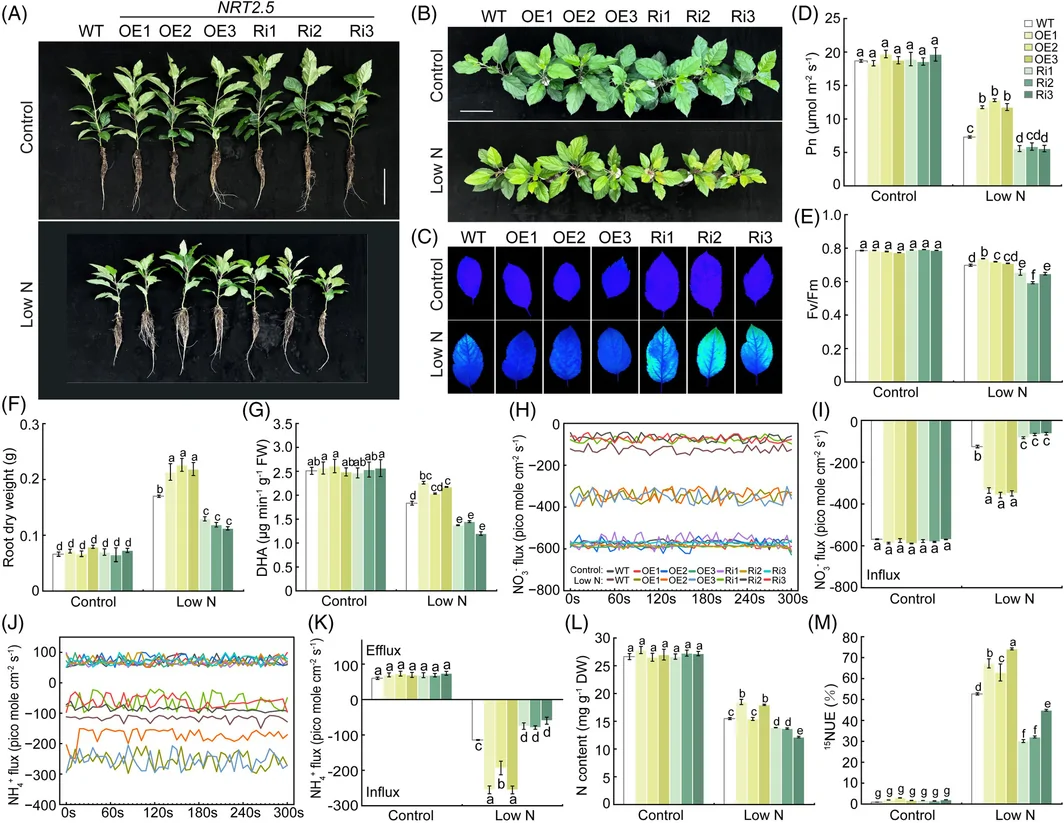
*NRT2.5* overexpression enhanced the tolerance of apple plants to low N stress. (A, B) Phenotypes of WT, *NRT2.5*‐OE and *NRT2.5*‐Ri apple lines after 30 days of cultivation under control or low N conditions. Scale bar = 10 cm. (C) Chlorophyll fluorescence images. (D) Net photosynthesis (Pn). (E) Fv/Fm ratio. (F) Root dry weight. (G) Root activity DHA. (H, I) Effects of low N stress on net NO_3_^−^ flux in different lines. (J, K) Effects of low N stress on net NH_4_^+^ flux in different lines. (L) N content. (M) NUE. Data are mean ± SD. According to one‐way ANOVA and Duncan multiple range test, different letters in I and K represent significant differences among different lines under control and low N stress, respectively, and in others represent significant differences among all the collected data (*P* < 0.05). WT, wild type, here we used GL‐3 apple (*Malus domestic*), which was also used as explants in generating transgenic apple plants; OE1/2/3, *NRT2.5*‐overexpression line 1/2/3; Ri1/2/3, *NRT2.5*‐RNA interference line 1/2/3.

As the nitrate transporter, we also used NMT to measure NO_3_^−^ and NH_4_^+^ fluxes in WT and *NRT2.5* transgenic apple roots. Under low N stress, the NO_3_^−^ influx was significantly higher in OE plants than in WT plants, and lowest in Ri plants (Figure 6H,I). A similar trend was observed for the NH_4_^+^ flux under low N stress (Figure 6J,K). The difference in the net influx made the total N content significantly higher in *NRT2.5*‐OE plants than in WT, and lower in *NRT2.5*‐Ri lines compared with WT plants (Figure 6L). The low N treatment significantly increased NUE in all lines. However, the NUE of OE1, OE2, and OE3 plants was 1.28‐, 1.19‐, and 1.41‐fold higher than that in WT plants, respectively, whereas NUE in Ri1, Ri2, and Ri3 plants was reduced to 0.57‐, 0.61‐, and 0.85‐fold of WT levels (Figure 6M). These results indicate that *NRT2.5* positively regulates NUE in apple plants under low N stress.

### AMT1.1 positively regulates the NUE of apple plants under low N stress

AMT1.1 was also localized in the cytoplasm (Figure S5B), and its expression level in leaves increased by approximately 2.5‐fold at 3 days of low N treatment, while in roots it increased by approximately 6‐fold at 5 days of low N treatment (Figure S5A). To better understand the role of *AMT1.1* under low N stress, we generated three transgenic apple lines overexpressing *AMT1.1* (OE1, −2, and −3, Figure S1G,H). *AMT1.1* overexpression did not affect apple plant growth; however, under low N stress, leaf yellowing and reddening were less severe in OE plants than in WT plants, and OE plants developed stronger root systems than WT plants (Figure 7A,B). Subsequently, we measured the chlorophyll content of all lines and found that it decreased in all plants under low N stress, whereas all OE lines maintained significantly higher chlorophyll content than WT plants (Figure 7D). Under low N stress, the Pn values of OE lines were significantly higher than those of WT lines (Figure 7E). Additionally, the Fv/Fm values of all plants decreased significantly under low N stress, but the Fv/Fm values of OE plants remained higher than those of WT plants (Figure 7F), and a similar trend was observed in the chlorophyll fluorescence images (Figure 7C). The *AMT1.1‐*OE plants also exhibited stronger root systems, similar to those of *NRT2.5*‐OE plants. Accordingly, root length and root dry weight were significantly greater in *AMT1.1*‐OE lines than in WT plants (Figure 7G,H), and root activity (indicated by DHA value) was also higher in *AMT1.1*‐OE lines than in WT plants under low N stress (Figure 7I).

**Figure 7:**
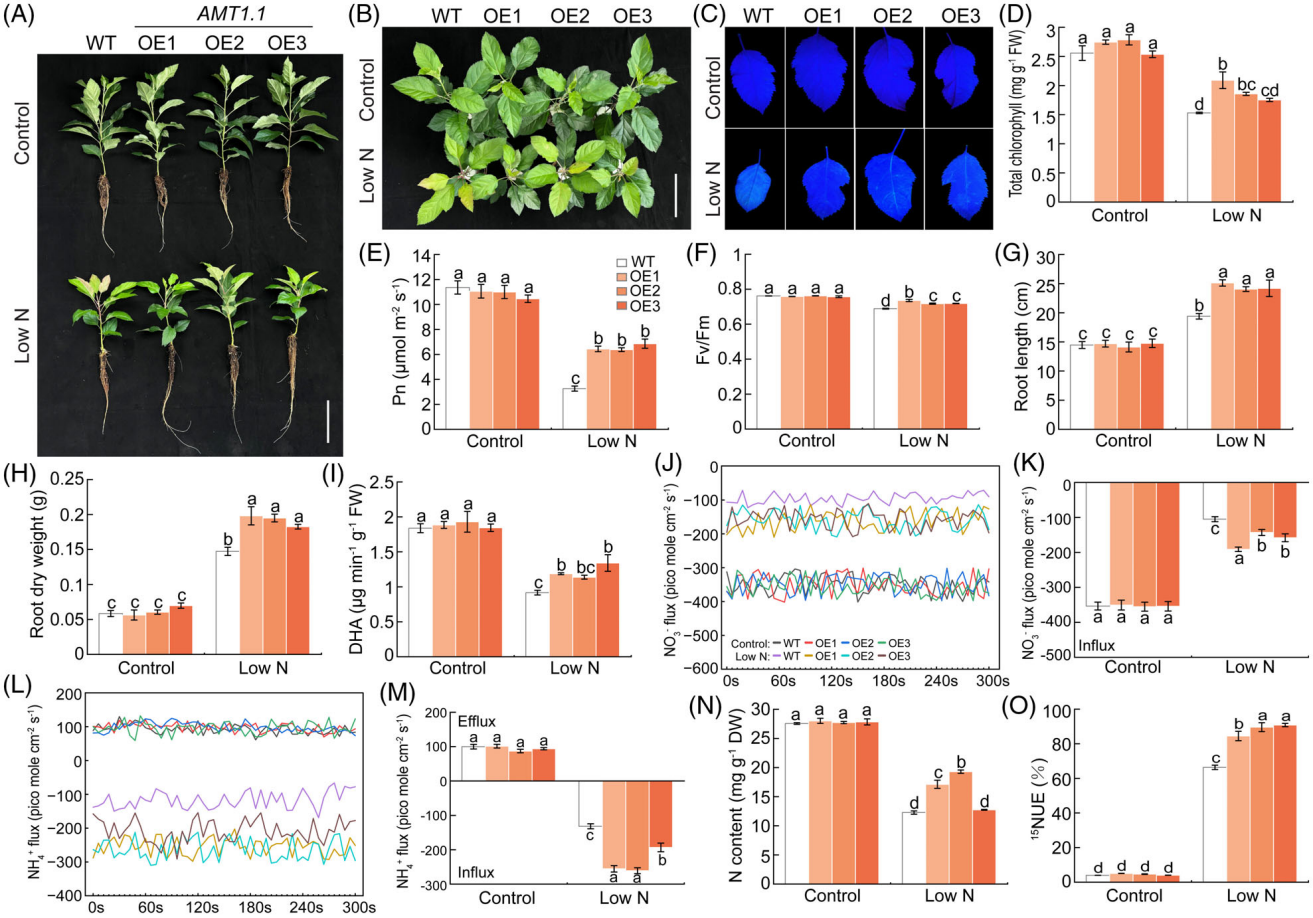
*AMT1.1* overexpression enhanced the tolerance of apple plants to low N stress. (A, B) Phenotypes of WT and *AMT1.1*‐OE apple lines after 30 days of cultivation under control or low N conditions. Scale bar = 10 cm. (C) Chlorophyll fluorescence images. (D) Total chlorophyll. (E) Net photosynthesis (Pn). (F) Fv/Fm ratio. (G) Root length. (H) Root dry weight. (I) DHA. (J, K) Effects of low N stress on net NO_3_^−^ flux in different lines. (L, M) Effects of low N stress on net NH_4_^+^ flux in different lines. (N) N content. (O) NUE. Data are mean ± SD. According to one‐way ANOVA and Duncan's multiple range test, different letters in K and M represent significant differences among different lines under control and low N stress, respectively, and in others represent significant differences among all the collected data (*P* < 0.05). WT, wild type, here, we used ‘GL‐3’ apple (*Malus domestica)*, which was also used as explants in generating transgenic apple plants; OE1/2/3, *AMT1.1*‐overexpression line 1/2/3.

We further investigated the role of *AMT1.1* in N uptake using NMT technology to measure NO_3_^−^ and NH_4_^+^ fluxes in the roots of each line. The results showed that under control conditions, there were no significant differences in NO_3_^−^ uptake rates among the lines. However, under low N conditions, the NO_3_^−^ uptake rates in OE plants were significantly higher than those in WT plants (Figure 7J,K). Additionally, under low N conditions, NH_4_^+^ uptake rates in OE plants were also significantly higher than those in WT plants (Figure 7L,M). The increased N uptake resulted in significantly higher total N content in *AMT1.1*‐OE lines compared with WT plants under low N stress (Figure 7N). When NUE was measured, the NUE of OE plants was significantly higher than that of WT plants, with OE1, OE2, and OE3 exhibiting NUE values that were 1.27‐, 1.35‐, and 1.37‐fold higher than those of WT plants under low N stress, respectively (Figure 7O). These results indicate that *AMT1.1* is a positive regulator of NUE and the low N stress response in apple plants.

### SnRK2.10 collaborates with the N transporters in regulating the NUE of apple plants under low N stress

To further determine the effect of the combined action of *SnRK2.10* and the two N transporters, *NRT2.5* and *AMT1.1*, on low N stress, we first suppressed the expression of *NRT2.5* in *SnRK2.10‐Ri* plants via VIGS (Figure S6A). When exposed to low N stress, suppression of *NRT2.5* further reduced the stress tolerance of *SnRK2.10*‐Ri plants; leaves of *SnRK2.10*‐Ri/*NRT2.5‐*Ri plants showed more severe chlorosis and redness than those of *SnRK2.10*‐Ri and WT plants (Figure 8A). Compared with *SnRK2.10*‐Ri plants, *SnRK2.10‐*Ri/*NRT2.5*‐Ri plants had lower Fv/Fm values, and chlorophyll fluorescence imaging showed a similar trend (Figure 8C,D). In addition, root growth of *SnRK2.10*‐Ri plants was further inhibited by suppression of *NRT2.5* (Figure 8B, *SnRK2.10‐*Ri/*NRT2.5*‐Ri vs. *SnRK2.10‐*Ri). DHA values were significantly lower in *SnRK2.10‐*Ri/*NRT2.5*‐Ri plants than in *SnRK2.10‐*Ri plants (Figure 8E). We also measured the flow rates of NO_3_^−^ and NH_4_^+^ ions in each line and found that both their absorption rates in *SnRK2.10*‐Ri/*NRT2.5*‐Ri plants were lower than those in *SnRK2.10*‐Ri plants under low N stress (Figure 8F–I). The total N content in *SnRK2.10*‐Ri/*NRT2.5*‐Ri plants was significantly lower than that in *SnRK2.10*‐Ri plants (Figure 8J), and the NUE of *SnRK2.10*‐Ri/*NRT2.5*‐Ri plants was significantly lower than that of *SnRK2.10*‐Ri plants under low N stress (Figure 8K).

**Figure 8:**
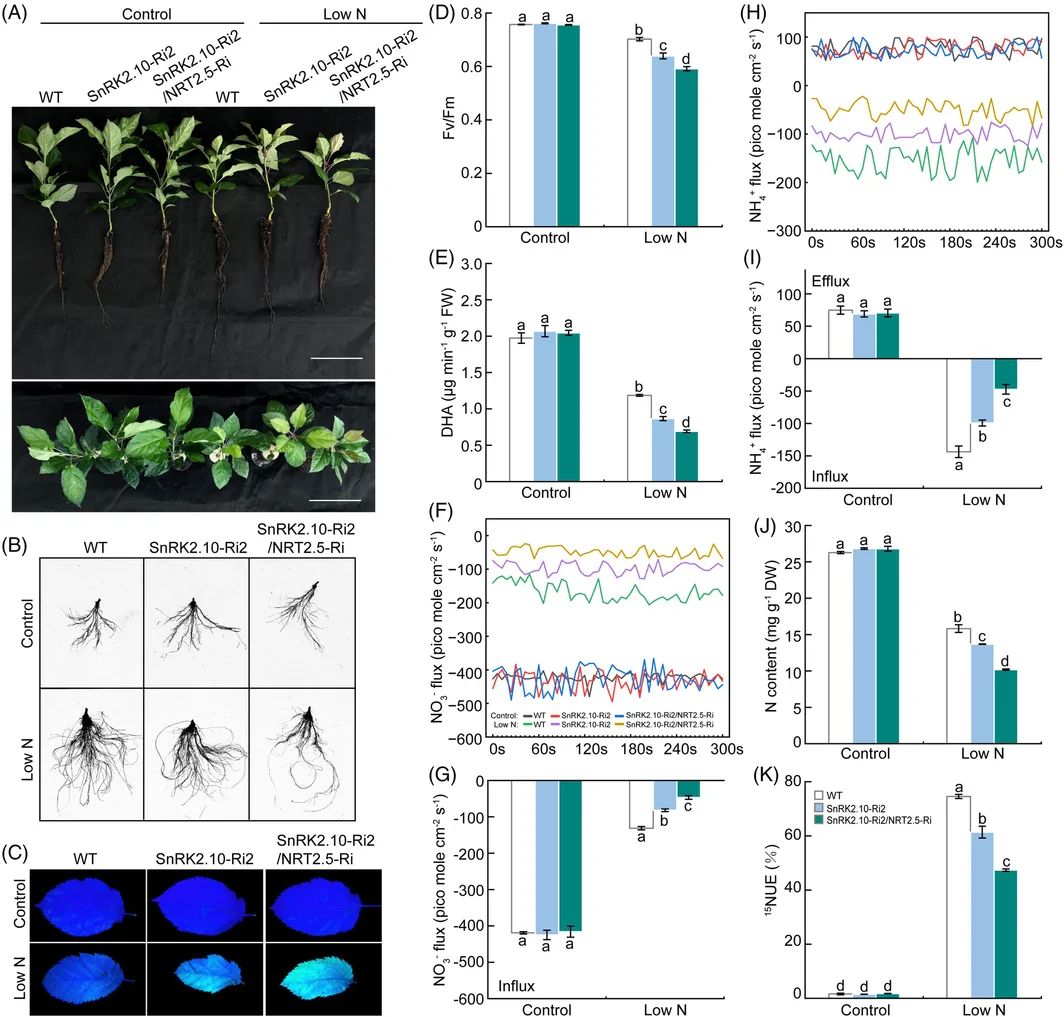
*SnRK2.10* and *NRT2.5* cooperatively enhanced the tolerance of apple plants to low nitrogen stress. (A) Phenotypes of *SnRK2.10*‐Ri apple plants with silenced *NRT2.5* mediated by transient transformation under low N stress. Scale bar = 10 cm. (B) Root system architecture scan diagram. (C) Chlorophyll fluorescence images. (D) Fv/Fm ratio. (E) DHA. (F, G) Effects of low N stress on net NO_3_^−^ flux in different lines. (H, I) Effects of low N stress on net NH_4_^+^ flux in different lines. (J) N content. (K) NUE. Data are mean ± SD. According to one‐way ANOVA and Duncan multiple range test, different letters in G and I represent significant differences among different lines under control and low N stress, respectively, and in others represent significant differences among all the collected data (*P* < 0.05).

We then generated *SnRK2.10*‐OE/*AMT1.1‐*OE plants by transiently overexpressing *SnRK2.10* in *AMT1.1‐*OE plants (Figure S6B). When exposed to low N stress, overexpressing both *SnRK2.10* and *AMT1.1* further enhanced the stress tolerance of apple plants than overexpressing *AMT1.1* alone; leaves of *SnRK2.10*‐OE/*AMT1.1*‐OE plants showed less chlorosis and redness than those of *AMT1.1*‐OE and WT plants (Figure 9A). Compared with *AMT1.1*‐OE plants, *SnRK2.10*‐OE/*AMT1.1*‐OE plants had higher Fv/Fm values, and chlorophyll fluorescence imaging showed a similar trend under low N stress (Figure 9C,D). In addition, root scanning showed that, under low N stress, co‐overexpression of *SnRK2.10* and *AMT1.1* further enhanced root growth of apple plants compared with *AMT1.1*‐OE and WT plants (Figure 9B). DHA measurements revealed that *SnRK2.10*‐OE/*AMT1.1*‐OE plants had significantly enhanced root activity (Figure 9E). We also measured the flow rates of NO_3_^−^ and NH_4_^+^ ions in each line and found that both their absorption rates in *SnRK2.10*‐OE/*AMT1.1*‐OE plants were higher than those in *AMT1.1*‐OE plants (Figure 9F–I). The total N content in *SnRK2.10*‐OE/*AMT1.1*‐OE plants was significantly higher than that in *AMT1.1*‐OE plants (Figure 9J), and the NUE of *SnRK2.10*‐OE/*AMT1.1*‐OE plants was also significantly higher than that of *AMT1.1*‐OE plants under low N stress (Figure 9K).

**Figure 9:**
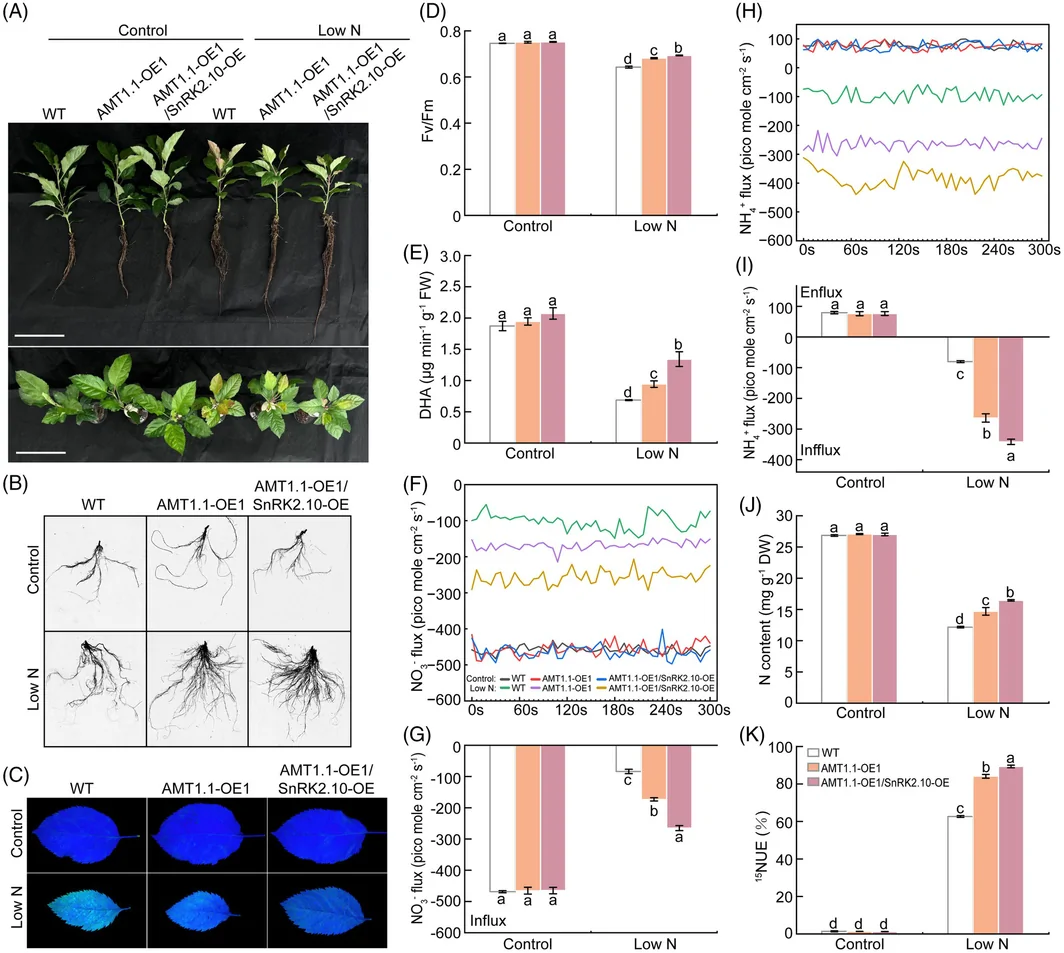
*SnRK2.10* and *AMT1.1* cooperatively enhanced the tolerance of apple plants to low nitrogen stress. (A) Phenotypes of *AMT1.1*‐OE apple plants with overexpressed *SnRK2.10* mediated by transient transformation under low N stress. Scale bar = 10 cm. (B) Root system architecture scan diagram. (C) Chlorophyll fluorescence images. (D) Fv/Fm ratio. (E) DHA. (F, G) Effects of low N stress on net NO_3_^−^ flux in different lines. (H, I) Effects of low N stress on net NH_4_^+^ flux in different lines. (J) N content. (K) NUE. Data are mean ± SD. According to one‐way ANOVA and Duncan multiple range test, different letters in G and I represent significant differences among different lines under control and low N stress, respectively, and in others represent significant differences among all the collected data (*P* < 0.05).

Taken together, these results suggest that the interaction between SnRK2.10 and the N transporters (NRT2.5 and AMT1.1) functions effectively in apple plants under low N stress. They collaboratively promoted NUE under low N stress, probably by enhancing N transport.

## DISCUSSION

Nitrogen is an essential macronutrient that plays a critical role in plant growth and development. N deficiency can reduce seed yield and chlorophyll content, cause leaf chlorosis, accelerate senescence, and impair overall plant growth, resulting in yield loss (Fu et al., 2022). The Loess Plateau is the largest apple‐producing region in China, the low NUE of apple varieties severely limits yield and fruit quality here. Functional genomic studies have revealed a number of genes involved in low N responses or the NUE regulation in apple plants. For instance, the transcription factor MdNLP7 promotes N uptake and assimilation by activating the expression of *MdNIA2* and *MdNRT1.1* (Feng et al., 2022). The *miR164*‐*MhNAC1* module plays a role in the N uptake of apple rootstocks, as well as in the regulation of citric acid secretion in roots (Wang, Xu, et al., 2024). The *SnRKs* members play important roles in enhancing plant tolerance to various stresses. For example, *CiPawSnRK2.1* deletion significantly reduces salt tolerance in walnut seedlings (Wang, Zhou, et al., 2024), *AcSnRK2.4* positively regulates heat stress responses in kiwifruit (Wang et al., 2025), and *TaSnRK2.4‐B* negatively regulates pathogen resistance through an ABA‐dependent pathway (Jiang et al., 2022). Our previous studies demonstrated that overexpression of *SnRK2.10* in *Arabidopsis* and apple enhances tolerance to drought and salt stress (Shao et al., 2019; Ye et al., 2022). Here, we further investigated the role of *SnRK2.10* in regulating NUE in apple plants. The low N treatment induced *SnRK2.10* expression in both roots and leaves (Figure 1). Overexpression of *SnRK2.10* significantly enhanced low N stress tolerance and NUE (Figure 2), whereas its suppression reduced low N tolerance and NUE in apple plants (Figure 2), indicating that *SnRK2.10* plays a positive role in regulating NUE under low N stress.

Under N‐deficient conditions, plants develop deeper and more extensive root systems to enhance N uptake, representing an important adaptive strategy (Aggarwal et al., 2025; Ju et al., 2015; Rasmussen et al., 2015; Smoczynska et al., 2022). *OsNAR2.2* enhances rice resistance to N starvation by promoting root growth and N uptake (Xu et al., 2020). *PpNrt2.1* and *PpNrt2.2* are specifically expressed in the roots of peach trees and are involved in the absorption of nitrate in the roots of peach trees (Nakamura et al., 2007). In this study, we found that plants overexpressing *SnRK2.10* exhibited more developed root systems and higher root vitality under low N stress (Figure 2), suggesting that *SnRK2.10* may also have enhanced N uptake efficiency. The integrated multi‐omics analysis indicated that SnRK2.10 may regulate N transport in response to low N (Figure 3). Further analysis confirmed that SnRK2.10 directly interacts with two N transport proteins, NRT2.5 (nitrate transporter) and AMT1.1 (ammonium transporter) (Figure 4). N uptake capacity is closely associated with NUE, and enhancing N uptake is a key strategy for improving crop NUE. For instance, *OsNRT1.1A* overexpression in rice significantly increased NUE and yield and also shortened the maturation period (Wang et al., 2018). Polymorphisms in *OsNRT1.1B* may lead to functional differences in NUE improvement in rice (Siangliw et al., 2024). Coordinated regulation of *BnNRT1.5* and *BnNRT1.8* promotes nitrate distribution to aboveground tissues in rapeseed‐type oil crops and improves NUE (Han et al., 2016). The ammonium transporter genes *BcAMT1s* differentially regulate the absorption and accumulation of NH_4_^+^ and NO_3_^−^, thereby influencing plant NUE (Straub et al., 2017). Although NRT2.5 and AMT1.1 are annotated as N transporters, their biological roles in apple under low N stress remain unclear. Therefore, we generated overexpression and silencing lines for these two genes for functional analysis. Overexpression of *NRT2.5* enhanced plant tolerance to low N stress, whereas silencing of *NRT2.5* reduced low N stress tolerance (Figure 6). In addition, overexpression of *AMT1.1* also enhanced low N stress tolerance (Figure 7), consistent with previous findings. Ion flux analysis showed that *NRT2.5* expression significantly affects NO_3_^−^ uptake and transport (Figure 6), while *AMT1.1* overexpression promotes NH_4_^+^ influx under low N stress (Figure 7). These results are consistent with the known functions of NRTs and AMTs, indicating that *NRT2.5* and *AMT1.1* improve NUE in transgenic plants by regulating NO_3_^−^ and NH_4_^+^ uptake.

SnRK2.10 interacted with both NRT2.5 and AMT1.1, suggesting that it may also affect N uptake to regulate NUE in apple plants. Ion flux analysis in *SnRK2.10* transgenic plants showed that, under low N stress conditions, plants overexpressing *SnRK2.10* exhibited higher NO_3_^−^ and NH_4_^+^ absorption than WT plants (Figure 5), preliminarily confirming that *SnRK2.10* has a regulatory role in N uptake. As a member of the SnRK2 protein kinase family, SnRK2.10 is likely to regulate the activity of its interacting proteins through phosphorylation. The protein kinases, such as CIPK23, regulate AMTs and thereby influence NH_4_^+^ and NO_3_^−^ uptake and accumulation (Straub et al., 2017). Similarly, the protein kinase OsACTPK1 mediates phosphorylation of OsAMT1 to prevent excessive ammonium accumulation in plants (Beier et al., 2018). Therefore, we speculate that SnRK2.10 may also phosphorylate NRT2.5 and AMT1.1 to alter their activity, stability, or subcellular localization, thereby optimizing transport function and ultimately enhancing plant NUE. The transport activity of NRTs is closely associated with phosphorylation at specific sites, enabling plants to adapt to different N conditions. When external nitrate levels decrease, phosphorylation of T101 converts NRT1.1 from a low‐affinity to a high‐affinity transporter, thereby regulating auxin flux and nitrate signal transduction during root growth (Bouguyon et al., 2016; Zhang et al., 2019). In contrast, phosphorylation of S585 markedly weakens its nitrate transport capacity (Su et al., 2021). Phosphorylation of T98 in NRT1.4 similarly reduces its transport activity (de los Ríos et al., 2021). When external nitrate levels increase, phosphorylation of S501 inhibits the transport activity of NRT2.1 and reduces high‐affinity nitrate uptake by roots (Jacquot et al., 2020). The transport activity of AMTs is also tightly regulated by phosphorylation at different sites. Mutation of T460 of AtAMT1.1 in *Arabidopsis thaliana* abolishes ammonium uptake, and similar effects can be observed for corresponding mutations in AtAMT1.2 (T472) and AtAMT1.3 (T464). These findings indicate that phosphorylation may be a conserved way of regulating the activity of these transporters (Neuhäuser et al., 2007; Wu et al., 2019). Constrained by technical challenges in phosphorylation experiments, we could not fully confirm the phosphorylation of NRT2.5 (or AMT1.1) by SnRK2.10. However, we constructed two kinds of co‐transgenic apple plants and analyzed their responses to low N stress, as well as their NUE, thus verifying the biological function of the interaction between SnRK2.10 and the two transporters (Figure S6). Under low N stress, overexpressing both *SnRK2.10* and N transporter gene (*AMT1.1*) further enhanced the stress tolerance of apple plants compared with overexpressing *AMT1.1* alone (Figure 9); while suppressing the expression of the N transporter gene (*NRT2.5*) in *SnRK2.10‐*OE plants significantly attenuated the low N stress tolerance of apple plants (Figure 8). These suggested that *SnRK2.10* positively regulates plant growth and NUE under low N stress, probably partially by the direct interaction with key N transporters.

Naturally, the present work is not without any limitations. First, the results relied on OE and Ri plants, although their phenotypes were consistent with their tolerance, they were not enough to resolve whether the observed effects are direct or mediated through broader changes in plant metabolism or growth. This is also a common issue that can be observed in current pomology studies (Rahmati et al., 2018; Kumar et al., 2021; Habibi et al., 2023; Liu et al., 2023). Given that the molecular changes typically occur earlier than physiological responses, we preferred to believe that the interaction between SnRK2.10 and NRT2.5 (or AMT1.1) helps to enhance N uptake, and ultimately improves apple growth under low N stress. Second, we overemphasized the consistency in measured indicators and ignored the unique functional characteristics of each gene, leading to some overlap in the functional characterization of *SnRK2.10*, *NRT2.5*, and *AMT1.1*. SnRK2.10 is a kinase, while the other two are N transporters, there should be different manner when they execute their biological function. Third, the synergistic effects of SnRK2.10 and NRT2.5 (or AMT1.1) have been proposed but have not been fully elucidated, since we only employed two kinds of co‐transgenic apple materials. The major technical challenge is that stable genetic transformation of woody species such as apple is highly time‐consuming and technically demanding. This limitation prevented us from more precisely separating the individual effects of *SnRK2.10*, *NRT2.5*, and *AMT1*.1 from their combined effects. The last one, the controlled conditions setting in the present work, allowed us to clearly evaluate the function of *SnRK2.10* and its interaction with NRT2.5/AMT1.1 in regulating N uptake and NUE, they cannot fully represent field settings or more complex soil environments. In particular, soil nitrogen availability, microbial activity, root‐soil interactions, seasonal environmental fluctuations, and orchard management practices may all influence N uptake and NUE of apple plants (Cao et al., 2019; Liang et al., 2025; Zhao et al., 2020). Collectively, these shortcomings offer valuable insights for our subsequent work, helping us achieve more robust research work.

## CONCLUSIONS

In summary, this study demonstrated that *SnRK2.10* was induced by low N stress in both roots and leaves, and that it positively regulated NUE and low N adaptive responses in apple plants. The omics analyses revealed that *SnRK2.10* modulated N metabolism pathways and suggested that N transporters contribute to its positive regulatory function. Further assays confirmed that SnRK2.10 interacts with the N transporters NRT2.5 and AMT1.1. Both transporters acted as positive regulators of N uptake and NUE in apple plants. In addition, NO_3_^−^ and NH_4_^+^ fluxes in roots of *SnRK2.10* transgenic apple lines were also significantly changed under low N stress (Figure 10). Overall, these results indicate that *SnRK2.10* positively regulates NUE in apple plants, most likely via functional cooperation with NRT2.5 and AMT1.1. Additional genetic evidence is required to fully clarify their individual and combined effects. Nevertheless, *SnRK2.10* is a promising target for genetic engineering to improve NUE in apple.

**Figure 10:**
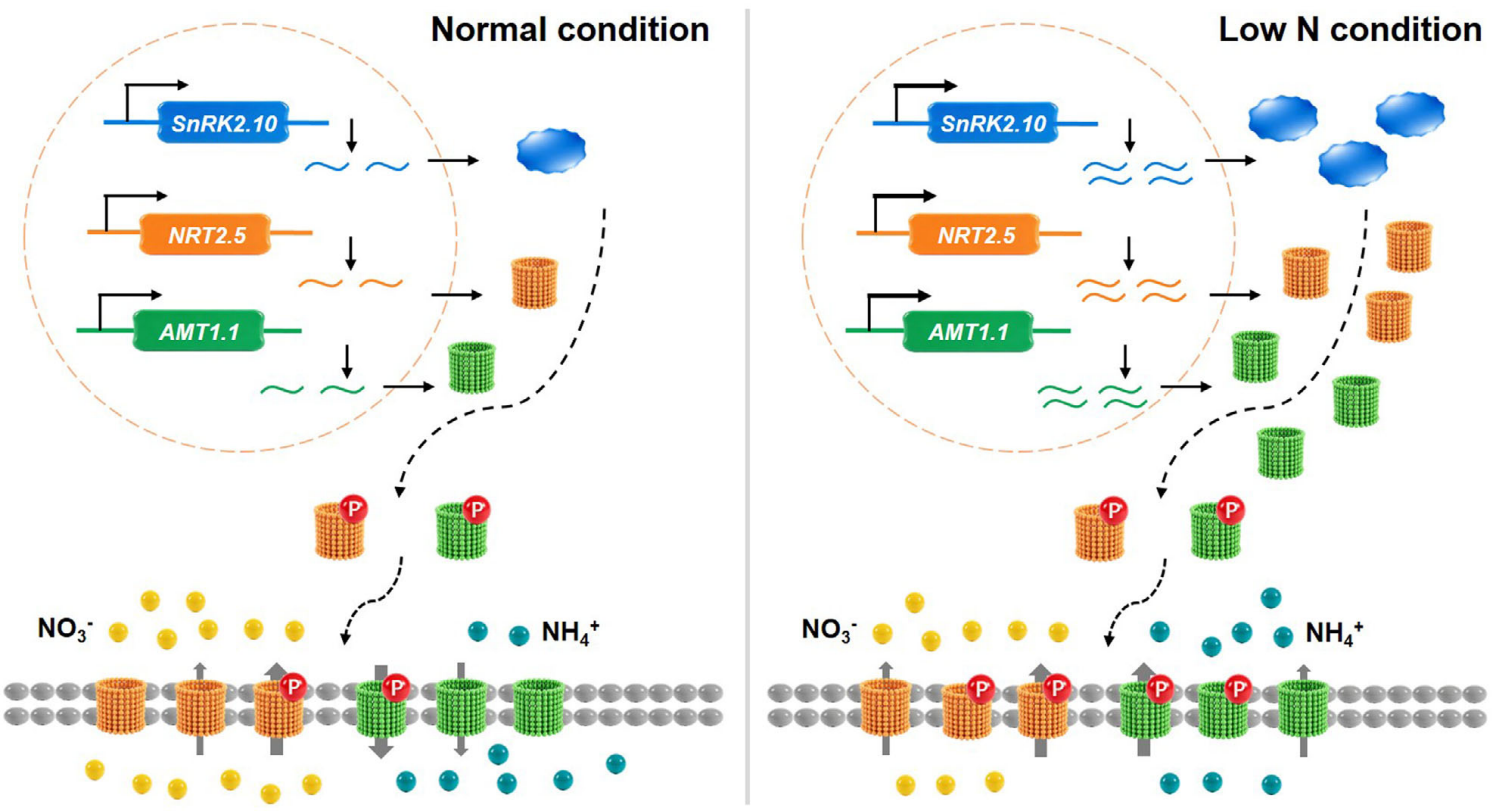
A working model demonstrating the role of *SnRK2.10* in the response of apple to low N stress. Low N stress induces the expression of *SnRK2.10, NRT2.5*, and *AMT1.1*. SnRK2.10 can interact with both NRT2.5 and AMT1.1, and this interaction probably affects the transporter activity of NRT2.5 and AMT1.1. The transport rates of both NO_3_^−^ and NH_4_^+^ in the *SnRK2.10*‐OE plants are enhanced under low N stress, leading to continued growth of the OE plants. Collectively, *SnRK2.10* positively regulates the tolerance of apple plants to low N stress, probably via synergistic function with the N transporter NRT2.5 and AMT1.1. Note: the drawing elements in the model are generated with the help of AI (Doubao). For instance, the barrel‐shaped transporters, the spheres with different colors, the rectangles with different gene names, and the irregular ellipse we used to mark the SnRK2.10 protein.

## MATERIALS AND METHODS

### Plant materials and treatments

‘GL‐3’, a cultivar derived from ‘Royal Gala’ apple (*Malus domestica*), was used as the wild‐type (WT) plant in genetic transformation, transient expression, and virus‐induced gene silencing (VIGS). *Nicotiana benthamiana* served as a heterologous expression system.

For the low N stress treatments, GL‐3 and transgenic apple plants were grown on rooting medium for 35 days and then transplanted into plastic pots (8 × 8 cm) containing a substrate of vermiculite:perlite:soil (3:1:1, v/v/v). Plants were maintained in a controlled growth chamber under cool white light (16 h light/8 h dark, 8000 lx). After 1 month, uniformly growing apple plants were selected for hydroponic low‐N treatment, following the previously described methods (Jia et al., 2022; Li et al., 2022). After a 7‐days pre‐culture, both WT and transgenic plants were randomly assigned to 0.02 mM N (Low N treatment) or 6 mM N (Control) for 30 days.

### Genetic transformation of apple plants

The *SnRK2.10* overexpression lines (OE1 and OE2) were previously generated by Shao et al. (2019). When generating transgenic apple lines overexpressing *NRT2.5* (or *AMT1.1*), the full coding sequence (CDS) of *NRT2.5* (or *AMT1.1*) was cloned into pCambia2300. Selected *SnRK2.10* and *NRT2.5* fragments were cloned into the phellsgate vector to generate RNAi constructs. The recombinant plasmids were introduced into *Agrobacterium* strain GV3101. Stable and transient transformations were performed following established protocols (Liu et al., 2025; Zhang et al., 2024). All the primers were listed in Table S1.

### In situ hybridization experiment

The *in situ* hybridization of *SnRK2.10* and *NRT2.5* was performed in apple tissues with minor modifications according to the previously described methods (Zhang et al., 2023). Roots, stems, and leaves were harvested and immediately fixed in FAA solution. Samples were decolorized, paraffin‐embedded, and sectioned. Sections were incubated at 37°C for 8–12 h, deparaffinized with xylene, and rehydrated using graded ethanol and DEPC‐treated water. Finally, the sections were then digested with 1 μg/mL proteinase K at 37°C, hybridized with probes at 42°C overnight, rinsed with washing buffer at 23°C and 65°C, and stored in TE buffer.

### Quantitative PCR (qPCR) analysis

The total RNA was extracted using a Wolact Plant RNA Isolation Kit (Wolact, Hong Kong, China). The first‐strand cDNA was synthesized from 1 μg of the total RNA using the RevertAid First Strand cDNA Synthesis Kit (Thermo Scientific, Waltham, MA, USA). The qPCR assays were conducted using SYBR Green qPCR Kits (Takara, Tokyo, Japan) on an iQ5 Multicolor Detection System (Bio‐Rad Laboratories, Hercules, CA, USA), according to Zhang et al. (2023). All the primers were listed in Table S1.

### Subcellular localization

The pCAMBIA2300‐GFP vector was digested with *Bam*H I and *Sal* I, and the CDS of *SnRK2.10*, *NRT2.5*, and *AMT1.1* were cloned by homologous recombination. Activated *Agrobacterium* cultures were resuspended to an OD600 of 0.6 and infiltrated into the leaves of *Nicotiana benthamiana*. Fluorescence signals were observed using a confocal laser scanning microscope (TCS‐SP8 SR; Leica, Germany).

### Photosynthetic indicators

After 30 days of low N stress treatment, the chlorophyll content was determined following the previously described method (Ye et al., 2022). Briefly, 0.1 g of leaf tissue was homogenized in 4 mL of 80% acetone and incubated in darkness for 24 h. Absorbance was measured using a UV–Vis spectrophotometer (UV‐2600, Shimadzu, Japan). The net photosynthetic rate (Pn) was measured using a CIRAS‐3 portable photosynthesis system (Amesbury, MA, USA). Following 30 min of dark adaptation, chlorophyll fluorescence parameters were recorded using an IMAGING‐PAM fluorometer (WALZ, Nuremberg, Germany). Leaf fluorescence images were analyzed to evaluate photosystem damage under different treatments.

### Measurements of growth parameters, N concentration, and stable isotope δ^15^N‐related indicators

At the end of the low N treatment, five plants per group were harvested for growth analysis. Root length was measured from the plant base to the tip of the main root. Root activity (DHA) was determined using the DHA detection kit (Suzhou Comin Biotechnology Co., Ltd, Suzhou, China). The root architecture was scanned using the SNAPSCAN 310 scanner, and the images were generated using the WinRhizo image analysis system.

For the N measurements, samples were fixed at 105°C for 30 min and dried at 65°C for 4 days. The root dry weight was then measured. The dried tissues (the whole plant, including roots, stems, and leaves) were ground into fine powder for N analysis. N concentration was determined as described previously (Jia et al., 2022). The stable isotope δ^15^N was measured using an elemental analyzer (Flash EA 1112HT, Thermo Fisher Scientific, USA) coupled with a stable isotope mass spectrometer (Finnigan Delta V Advantage, Thermo Fisher Scientific, USA).

### Determination of flux of NO_3_ ^−^/NH_4_ ^+^

The net NO_3_^−^ /NH_4_^+^ flux was measured using non‐invasive micro‐test technology (NMT; Younger USA, LLC, Amherst, MA, USA). Flux measurements were performed approximately 20 μm from the root tip along the root axis. After 15 days of low N and control treatments, tender root tips were transferred to measurement solutions and equilibrated for 30 min. Ion fluxes were then recorded for 5 min and analyzed using imFluxes software (v2.0; Beijing Xuyue Technology Co., Ltd.). Three independent biological replicates were conducted, each comprising three randomly selected roots per line.

### RNA‐seq and proteomic analyses

Under the control conditions, leaf samples were collected from *SnRK2.10*‐OE2 and WT for transcriptomic analysis by Wuhan Seqhealth Tech Co., Ltd. Raw sequencing data were aligned to the apple reference genome (GDDH13_v1.1_GDR) using Base Calling. Quality control, transcriptome assembly, and quantification were performed using fastp (v0.23.0). Differentially expressed genes (DEGs) were identified using DESeq2, with significance thresholds of adjusted *P* < 0.05 and |fold change| ≥1.5.

In addition, the leaf samples were collected for proteomic analysis by Beijing Tsingke Biotech Co., Ltd. Raw mass spectrometry data were processed using MaxQuant (v1.6.15.0), and MS/MS spectra were searched against the Blast_Malus_domestica_3750_PR_20210319.fasta database containing 42 478 entries. Differentially expressed proteins (DEPs) were identified using DESeq2, with significance thresholds of adjusted *P* < 0.05 and |fold change| ≥1.2.

### Protein interaction assays

For yeast two‐hybrid (Y2H) assays, the pGBKT7 vector was digested with *Eco*R I and *Sal* I, and the *NRT2.5* CDS and *AMT1.1* CDS were inserted using homologous recombination, respectively. The pGADT7 vector was digested with *Bam*H I and *Eco*R I, and the *SnRK2.10* CDS was inserted into it. The resulting constructs were co‐transformed into the yeast strain Y2H‐Gold. Monoclonal colonies selected on SD/−Trp‐Leu medium were transferred to SD/−Trp‐Leu‐His‐Ade medium and incubated at 28°C for 3 days to assess yeast growth.

For the Split luciferase (Split‐LUC) complementation assays, the pCAMBIA1300‐n/cLUC vector was digested with *Kpn* I and *Sal* I. The *NRT2.5* CDS and *AMT1.1* CDS were cloned into pCAMBIA1300‐nLUC, respectively, and the *SnRK2.10* CDS was cloned into pCAMBIA1300‐cLUC via homologous recombination. Recombinant plasmids were transformed into *Agrobacterium tumefaciens* GV3101, and bacterial suspensions were adjusted to an OD600 of 1.0–1.2. Different combinations were infiltrated into *Nicotiana benthamiana* leaves, and luciferase activity was detected 48–72 h after infiltration using an *in vivo* imaging system (PlantView100).

For the pull‐down assay, the *SnRK2.10* CDS was cloned into pGEX4T‐1 with a GST tag, and the *NRT2.5* CDS and *AMT1.1* CDS were cloned into pET28a with a His tag, respectively. The recombinant plasmids were transformed into *Escherichia coli* BL21 (DE3), and protein expression was induced with IPTG at 4°C for 24 h. Proteins were purified according to the manufacturer's protocol (TianDiRenSheng, Smart‐Lifesciences, Jiangsu, China), and immunoblotting was performed using anti‐GST and anti‐His antibodies (Bioss, Beijing, China).

### Statistical analysis

All data are presented as mean ± SD. Statistical analyses were performed using one‐way analysis of variance (ANOVA) in SPSS 27.0. Differences among data were evaluated using Duncan's multiple range test, with statistical significance set at *P* < 0.05, marked with different lowercase letters.

## Author Contributions

FM and XG conceived the ideas; YY, ZZ, ZL, LL, XH and YD collected and analyzed the data; Ying Ye and Xiaoqing Gong wrote the manuscript; FM and XG revised the manuscript and provided financial support. All authors contributed critically to the drafts and gave final approval for publication.

## Conflicts of Interest

The authors declare that there are no conflicts of interest.

## Supporting information


**Figure S1.** Identification of transgenic apple plants. (A) Identification of the *SnRK2.10* RNAi lines via PCR. (B) qPCR analysis of *SnRK2.10* in the RNAi lines. (C) Identification of the *NRT2.5* overexpression lines via PCR. (D) qPCR analysis of *NRT2.5* in the overexpression lines. (E) Identification of the *NRT2.5* RNAi lines via PCR. (F) qPCR analysis of *NRT2.5* in the RNAi lines. (G) Identification of the *AMT1.1* overexpression lines via PCR. (H) qPCR analysis of *AMT1.1* in the overexpression lines. Data are mean ± SD. According to one‐way ANOVA and Duncan multiple range test, different letters represent significant differences between different plants (*P* < 0.05).

**Figure S2.** Analysis of DEGs in WT and *SnRK2.10* overexpression apple leaves. (A) Number of upregulated and downregulated differentially expressed genes (DEGs). (B) Cluster diagram of DEGs. (C) Mapman enrichment analyses between the WT and *SnRK2.10*‐OE. The colors in B indicate the expression of each DEG as determined by the average peak response area with Z‐score normalization. WT, wild type, here we used GL‐3 apple (*Malus domestic*), which was also used as explants in generating transgenic apple plants; OE, *SnRK2.10*‐overexpression line, here we used OE2.

**Figure S3.** Analysis of DEPs in WT and *SnRK2.10* overexpression apple leaves. (A) Number of upregulated and downregulated differentially expressed proteins (DEPs). (B) Cluster diagram of DEPs. (C) Mapman enrichment analyses between the WT and *SnRK2.10*‐OE. The colors in B indicate the expression of each DEP as determined by the average peak response area with Z‐score normalization. WT, wild type, here we used GL‐3 apple (*Malus domestic*), which was also used as explants in generating transgenic apple plants; OE, *SnRK2.10*‐overexpression line, here we used OE2.

**Figure S4.** Expression patterns and subcellular localization of apple NRT2.5. (A) The expression level of *NRT2.5* in the leaves and roots under low N stress. (B) Subcellular localization of apple NRT2.5. Scale bar = 100 μm; BF, bright field; GFP, green fluorescent protein. (C) *In situ* hybridization of *NRT2.5* in roots, stems, and leaves of GL‐3 apple (*Malus domestica*) under control conditions (CK) and under low N conditions (low N). ep, epidermis; xy, xylem; ph, phloem; pi, pith; co, cortex; cu, cuticle; ue, upper epidermis; le, lower epidermis; pa, palisade mesophyll cells; sp, spongy mesophyll cells; vb, vascular bundle; ve, vein. Scale bar = 20 μm. Data are mean ± SD. According to one‐way ANOVA and Duncan multiple range test, different letters represent significant differences between data (*P* < 0.05).

**Figure S5.** Expression patterns and subcellular localization of apple AMT1.1. (A) Subcellular localization of apple AMT1.1. Scale bar= 100 μm; BF, bright field; GFP, green fluorescent protein. (B) The expression level of *AMT1.1* under low N stress. Data are mean ± SD. According to one‐way ANOVA and Duncan multiple range test, different letters represent significant differences between data (*P* < 0.05).

**Figure S6.** Identification of transgenic apples. (A) qPCR identification of *SnRK2.10* ‐ Ri apple plants with silenced *NRT2.5*. (B) qPCR identification of *AMT1.1*‐ OE apple plants with overexpressed *SnRK2.10*. Data are mean ± SD. According to one‐way ANOVA and Duncan multiple range test, different letters represent significant differences between data (*P* < 0.05).

**Table S1.** The primers used in this study.

**Table S2.** The detailed information of the 17 DEGs and 6 DEPs showed in Figure 3.

## Data Availability

The omics data are available for limited sharing upon reasonable request, as appropriate.

## References

[tpj71139-bib-0001] Aggarwal, S. , Rathore, R.S. , Rakhi, R. , Kumari, S. , Singla‐Pareek, S.L. & Mustafiz, A. (2025) OsCEP8‐mediated abiotic stress response is associated with auxin and sugar homeostasis in plants. Environmental and Experimental Botany, 229. Available from: 10.1016/j.envexpbot.2024.106082

[tpj71139-bib-0002] Bajgain, P. , Russell, B. & Mohammadi, M. (2018) Phylogenetic analyses and in‐seedling expression of ammonium and nitrate transporters in wheat. Scientific Reports, 8, 7082. Available from: 10.1038/s41598-018-25430-8 PMC5935732 29728590

[tpj71139-bib-0003] Beier, M.P. , Obara, M. , Taniai, A. , Sawa, Y. , Ishizawa, J. , Yoshida, H. et al. (2018) Lack of ACTPK1, an STY kinase, enhances ammonium uptake and use, and promotes growth of rice seedlings under sufficient external ammonium. The Plant Journal, 93(6), 992–1006. Available from: 10.1111/tpj.13824 29356222

[tpj71139-bib-0004] Boudsocq, M. , Barbier‐Brygoo, H. & Laurière, C. (2004) Identification of nine sucrose nonfermenting 1‐related protein kinases 2 activated by hyperosmotic and saline stresses in Arabidopsis thaliana. Journal of Biological Chemistry, 279(40), 41758–41766. Available from: 10.1074/jbc.M405259200 15292193

[tpj71139-bib-0005] Bouguyon, E. , Perrine‐Walker, F. , Pervent, M. , Rochette, J. , Cuesta, C. , Benkova, E. et al. (2016) Nitrate controls root development through posttranscriptional regulation of the NRT1.1/NPF6.3 transporter/sensor. Plant Physiology, 172(2), 1237–1248. Available from: 10.1104/pp.16.01047 PMC5047109 27543115

[tpj71139-bib-0006] Cao, H. , Ning, L.F. , Xun, M. , Feng, F. , Li, P. , Yue, S.Q. et al. (2019) Biochar can increase nitrogen use efficiency of Malus hupehensis by modulating nitrate reduction of soil and root. Applied Soil Ecology, 135, 25–32. Available from: 10.1016/j.apsoil.2018.11.002

[tpj71139-bib-0007] Cheng, H.Y. , Wu, B.D. , Wang, S. , Wei, M. & Wang, C.Y. (2021) Nitrogen application and osmotic stress antagonistically affect wheat seed germination and seedling growth. International Journal of Phytoremediation, 23(12), 1289–1300. Available from: 10.1080/15226514.2021.1895715 33689505

[tpj71139-bib-0008] Coello, P. , Hey, S.J. & Halford, N.G. (2011) The sucrose non‐fermenting‐1‐related (SnRK) family of protein kinases: potential for manipulation to improve stress tolerance and increase yield. Journal of Experimental Botany, 62(3), 883–893. Available from: 10.1093/jxb/erq331 20974737

[tpj71139-bib-0009] Comadira, G. , Rasool, B. , Karpinska, B. , Morris, J. , Verrall, S.R. , Hedley, P.E. et al. (2015) Nitrogen deficiency in barley (Hordeum vulgare) seedlings induces molecular and metabolic adjustments that trigger aphid resistance. Journal of Experimental Botany, 66(12), 3639–3655. Available from: 10.1093/jxb/erv276 PMC4463806 26038307

[tpj71139-bib-0010] de los Ríos, L.M. , Corratgé‐Faillie, C. , Raddatz, N. , Mendoza, I. , Lindahl, M. , de Angeli, A. et al. (2021) The Arabidopsis protein NPF6.2/NRT1.4 is a plasma membrane nitrate transporter and a target of protein kinase CIPK23. Plant Physiology and Biochemistry, 168, 239–251. Available from: 10.1016/j.plaphy.2021.10.016 34656860

[tpj71139-bib-0011] Dechorgnat, J. , Patrit, O. , Krapp, A. , Fagard, M. & Daniel‐Vedele, F. (2012) Characterization of the Nrt2.6 gene in Arabidopsis thaliana: a link with plant response to biotic and abiotic stress. PLoS One, 7(8). Available from: 10.1371/journal.pone.0042491 PMC3413667 22880003

[tpj71139-bib-0012] Feng, Z.Q. , Li, T. , Wang, X. , Sun, W.J. , Zhang, T.T. , You, C.X. et al. (2022) Identification and characterization of apple MdNLP7 transcription factor in the nitrate response. Plant Science, 316. Available from: 10.1016/j.plantsci.2021.111158 35151440

[tpj71139-bib-0013] Fu, Y.F. , Yang, X.Y. , Zhang, Z.W. & Yuan, S. (2022) Synergistic effects of nitrogen metabolites on auxin regulating plant growth and development. Frontiers in Plant Science, 13, 1098787. Available from: 10.3389/fpls.2022.1098787 PMC9807920 36605959

[tpj71139-bib-0014] Gonçalves, A.Z. , Oliveira, P.M.R. , Neto, A.A.C. & Mercier, H. (2020) Thinking of the leaf as a whole plant: how does N metabolism occur in a plant with foliar nutrient uptake? Environmental and Experimental Botany, 178. Available from: 10.1016/j.envexpbot.2020.104163

[tpj71139-bib-0015] Habibi, F. , Liu, T. , Shahid, M.A. , Schaffer, B. & Sarkhosh, A. (2023) Physiological, biochemical, and molecular responses of fruit trees to root zone hypoxia. Environmental and Experimental Botany, 206. Available from: 10.1016/j.envexpbot.2022.105179

[tpj71139-bib-0016] Han, Y.L. , Song, H.X. , Liao, Q. , Yu, Y. , Jian, S.F. , Lepo, J.E. et al. (2016) Nitrogen use efficiency is mediated by vacuolar nitrate sequestration capacity in roots of *Brassica napus* . Plant Physiology, 170(3), 1684–1698. Available from: 10.1104/pp.15.01377 PMC4775117 26757990

[tpj71139-bib-0017] Huang, L.F. , Zhang, H.C. , Zhang, H.Y. , Deng, X.W. & Wei, N. (2015) HY5 regulates nitrite reductase 1 (NIR1) and ammonium transporter1;2 (AMT1;2) in Arabidopsis seedlings. Plant Science, 238, 330–339. Available from: 10.1016/j.plantsci.2015.05.004 PMC4719586 26259199

[tpj71139-bib-0018] Huché‐Thélier, L. , Boumaza, R. , Demotes‐Mainard, S. , Canet, A. , Symoneaux, R. , Douillet, O. et al. (2011) Nitrogen deficiency increases basal branching and modifies visual quality of the rose bushes. Scientia Horticulturae, 130(1), 325–334. Available from: 10.1016/j.scienta.2011.07.007

[tpj71139-bib-0019] Jacquot, A. , Chaput, V. , Mauries, A. , Li, Z. , Tillard, P. , Fizames, C. et al. (2020) New Phytologist, 228(3), 1038–1054. Available from: 10.1111/nph.16710 32463943

[tpj71139-bib-0020] Jia, X. , Wang, Q. , Ye, Y. , Li, T.T. , Sun, X. , Huo, L.Q. et al. (2022) *MdATG5a* positively regulates nitrogen uptake under low nitrogen conditions by enhancing the accumulation of flavonoids and auxin in apple roots. Environmental and Experimental Botany, 197. Available from: 10.1016/j.envexpbot.2022.104840

[tpj71139-bib-0021] Jiang, B.H. , Liu, Y.K. , Niu, H.L. , He, Y.Q. , Ma, D.F. & Li, Y. (2022) Mining the roles of wheat (Triticum aestivum) SnRK genes in biotic and abiotic responses. Frontiers in Plant Science, 13, 934226. Available from: 10.3389/fpls.2022.934226 PMC9280681 35845708

[tpj71139-bib-0022] Ju, C.X. , Buresh, R.J. , Wang, Z.Q. , Zhang, H. , Liu, L.J. , Yang, J.C. et al. (2015) Root and shoot traits for rice varieties with higher grain yield and higher nitrogen use efficiency at lower nitrogen rates application. Field Crops Research, 175, 47–55. Available from: 10.1016/j.fcr.2015.02.007

[tpj71139-bib-0023] Kiba, T. , Feria‐Bourrellier, A.B. , Lafouge, F. , Lezhneva, L. , Boutet‐Mercey, S. , Orsel, M. et al. (2012) The Arabidopsis nitrate transporter NRT2.4 plays a double role in roots and shoots of nitrogen‐Straved plants. Plant Cell, 24(1), 245–258. Available from: 10.1105/tpc.111.092221 PMC3289576 22227893

[tpj71139-bib-0024] Kumar, V.V.S. , Yadav, S.K. , Verma, R.K. , Shrivastava, S. , Ghimire, O. , Pushkar, S. et al. (2021) The abscisic acid receptor OsPYL6 confers drought tolerance to indica rice through dehydration avoidance and tolerance mechanisms. Journal of Experimental Botany, 72(4), 1411–1431. Available from: 10.1093/jxb/eraa509 33130892

[tpj71139-bib-0025] Lezhneva, L. , Kiba, T. , Feria‐Bourrellier, A.B. , Lafouge, F. , Boutet‐Mercey, S. , Zoufan, P. et al. (2014) The Arabidopsis nitrate transporter NRT2.5 plays a role in nitrate acquisition and remobilization in nitrogen‐starved plants. The Plant Journal, 80(2), 230–241. Available from: 10.1111/tpj.12626 25065551

[tpj71139-bib-0026] Li, D.Y. , Ding, Y.D. , Cheng, L. , Zhang, X.L. , Cheng, S.Y. , Ye, Y. et al. (2022) Target of rapamycin (TOR) regulates the response to low nitrogen stress via autophagy and hormone pathways in Malus hupehensis. Horticulture Research, 9. Available from: 10.1093/hr/uhac143 PMC9437726 36072834

[tpj71139-bib-0027] Li, J. , Li, Q. , Guo, N. , Xian, Q.L. , Lan, B. , Nangia, V. et al. (2024) Polyamines mediate the inhibitory effect of drought stress on nitrogen reallocation and utilization to regulate grain number in wheat. Journal of Experimental Botany, 75(3), 1016–1035. Available from: 10.1093/jxb/erad393 37813095

[tpj71139-bib-0028] Liang, Q. , Zhang, T.B. , Kuang, Y.X. , Jiliu, Y.T. , Cheng, Y. , Gao, W.Q. et al. (2025) Ryegrass intercropping with residue incorporation enhances apple yield and reduces nitrogen loss despite minimal water‐nitrogen competition on the loess plateau, China. Agriculture, Ecosystems & Environment, 389. Available from: 10.1016/j.agee.2025.109700

[tpj71139-bib-0029] Liu, H. , Du, B.Y. , Ma, X.C. , Wang, Y. , Cheng, N.N. & Zhang, Y.H. (2023) Overexpression of major latex protein 423 (NtMLP423) enhances the chilling stress tolerance in Nicotiana tabacum. Plant Science, 329. Available from: 10.1016/j.plantsci.2023.111604 36709884

[tpj71139-bib-0030] Liu, X.M. , Li, J. , Cao, H.F. , Tian, X.D. , Li, S.J. , Liu, C.Y. et al. (2025) Dopamine regulates its own synthesis via MdORG2 to improve low‐nitrogen tolerance in apple plants. Plant, Cell and Environment, 48(1), 470–488. Available from: 10.1111/pce.15153 39279496

[tpj71139-bib-0031] Lu, Y. , Sasaki, Y. , Li, X.W. , Mori, I.C. , Matsuura, T. , Hirayama, T. et al. (2015) ABI1 regulates carbon/nitrogen‐nutrient signal transduction independent of ABA biosynthesis and canonical ABA signalling pathways in Arabidopsis. Journal of Experimental Botany, 66(9), 2763–2771. Available from: 10.1093/jxb/erv086 PMC4986877 25795738

[tpj71139-bib-0032] Lv, M.L. , Dong, T.T. , Wang, J. & Zuo, K.J. (2022) Genome‐wide identification of nitrate transporter genes from *Spirodela polyrhiza* and characterization of *SpNRT1.1* function in plant development. Frontiers in Plant Science, 13. Available from: 10.3389/fpls.2022.945470 PMC9436390 36061775

[tpj71139-bib-0033] Ma, W.Y. , Li, J.J. , Qu, B.Y. , He, X. , Zhao, X.Q. , Li, B. et al. (2014) Auxin biosynthetic gene TAR2 is involved in low nitrogen‐mediated reprogramming of root architecture in Arabidopsis. The Plant Journal, 78(1), 70–79. Available from: 10.1111/tpj.12448 24460551

[tpj71139-bib-0034] Mazur, R. , Maszkowska, J. , Anielska‐Mazur, A. , Garstka, M. , Polkowska‐Kowalczyk, L. , Czajkowska, A. et al. (2021) The SnRK2.10 kinase mitigates the adverse effects of salinity by protecting photosynthetic machinery. Plant Physiology, 187(4), 2785–2802. Available from: 10.1093/plphys/kiab438 PMC8644180 34632500

[tpj71139-bib-0035] Nakamura, Y. , Umemiya, Y. , Masuda, K. , Inoue, H. & Fukumoto, M. (2007) Molecular cloning and expression analysis of cDNAs encoding a putative Nrt2 nitrate transporter from peach. Tree Physiology, 27(4), 503–510. Available from: 10.1093/treephys/27.4.503 17241992

[tpj71139-bib-0036] Neuhäuser, B. , Dynowski, M. , Mayer, M. & Ludewig, U. (2007) Regulation of NH4+ transport by essential cross talk between AMT monomers through the carboxyl tails. Plant Physiology, 143(4), 1651–1659. Available from: 10.1104/pp.106.094243 PMC1851830 17337531

[tpj71139-bib-0037] Ondzighi‐Assoume, C.A. , Chakraborty, S. & Harris, J.M. (2016) Environmental nitrate stimulates abscisic acid accumulation in Arabidopsis root tips by releasing it from inactive stores. The Plant Cell, 28(3), 729–745. Available from: 10.1105/tpc.15.00946 PMC4826012 26887919

[tpj71139-bib-0038] Perchlik, M. & Tegeder, M. (2018) Leaf amino acid supply affects photosynthetic and plant nitrogen use efficiency under nitrogen stress. Plant Physiology, 178(1), 174–188. Available from: 10.1104/pp.18.00597 PMC6130036 30082496

[tpj71139-bib-0039] Rahmati, M. , Mirás‐Avalos, J.M. , Valsesia, P. , Lescourret, F. , Génard, M. , Davarynejad, G.H. et al. (2018) Disentangling the effects of water stress on carbon acquisition, vegetative growth, and fruit quality of peach trees by means of the QualiTree model. Frontiers in Plant Science, 9, 3. Available from: 10.3389/fpls.2018.00003 PMC5788000 29416545

[tpj71139-bib-0040] Rasmussen, I.S. , Dresboll, D.B. & Thorup‐Kristensen, K. (2015) Winter wheat cultivars and nitrogen (N) fertilization‐effects on root growth, N uptake efficiency and N use efficiency. European Journal of Agronomy, 68, 38–49. Available from: 10.1016/j.eja.2015.04.003

[tpj71139-bib-0041] Robaglia, C. , Thomas, M. & Meyer, C. (2012) Sensing nutrient and energy status by SnRK1 and TOR kinases. Current Opinion in Plant Biology, 15(3), 301–307. Available from: 10.1016/j.pbi.2012.01.012 22305521

[tpj71139-bib-0042] Sadras, V.O. & Richards, R.A. (2014) Improvement of crop yield in dry environments: benchmarks, levels of organisation and the role of nitrogen. Journal of Experimental Botany, 65(8), 1981–1995. Available from: 10.1093/jxb/eru061 24638898

[tpj71139-bib-0043] Shao, Y. , Zhang, X.L. , van Nocker, S. , Gong, X.Q. & Ma, F.W. (2019) Overexpression of a protein kinase gene *MpSnRK2*.*10* from *Malus prunifolia* confers tolerance to drought stress in transgenic *Arabidopsis thaliana* and apple. Gene, 692, 26–34. Available from: 10.1016/j.gene.2018.12.070 30641216

[tpj71139-bib-0044] Siangliw, J.L. , Ruangsiri, M. , Theerawitaya, C. , Cha‐um, S. , Poncheewin, W. , Songtoasesakul, D. et al. (2024) Contrasting alleles of *OsNRT1.1b* fostering potential in improving nitrogen use efficiency in Rice. Plants, 13(20). Available from: 10.3390/plants13202932 PMC11510876 39458879

[tpj71139-bib-0045] Smoczynska, A. , Pacak, A. , Grabowska, A. , Bielewicz, D. , Zadworny, M. , Singh, K. et al. (2022) Excess nitrogen responsive HvMADS27 transcription factor controls barley root architecture by regulating abscisic acid level. Frontiers in Plant Science, 13, 950796. Available from: 10.3389/fpls.2022.950796 PMC9511987 36172555

[tpj71139-bib-0046] Song, J. , Sun, P.P. , Kong, W.A. , Xie, Z.Z. , Li, C.L. & Liu, J.H. (2022) SnRK2.4‐mediated phosphorylation of ABF2 regulates ARGININE *DECARBOXYLASE* expression and putrescine accumulation under drought stress. New Phytologist, 238(1), 216–236. Available from: 10.1111/nph.18526 36210523

[tpj71139-bib-0047] Straub, T. , Ludewig, U. & Neuhäuser, B. (2017) The kinase CIPK23 inhibits ammonium transport in *Arabidopsis thaliana* . The Plant Cell, 29(2), 409–422. Available from: 10.1105/tpc.16.00806 PMC5354196 28188265

[tpj71139-bib-0048] Su, H. , Wang, T. , Ju, C.F. , Deng, J.P. , Zhang, T.Q. , Li, M.J. et al. (2021) Abscisic acid signaling negatively regulates nitrate uptake via phosphorylation of NRT1.1 by SnRK2s in *Arabidopsis* . Journal of Integrative Plant Biology, 63(3), 597–610. Available from: 10.1111/jipb.13057 33331676

[tpj71139-bib-0049] Szymanska, K.P. , Polkowska‐Kowalczyk, L. , Lichocka, M. , Maszkowska, J. & Dobrowolska, G. (2019) SNF1‐related protein kinases SnRK2.4 and SnRK2.10 modulate ROS homeostasis in plant response to salt stress. International Journal of Molecular Sciences, 20, 1. Available from: 10.3390/ijms20010143 PMC6337402 30609769

[tpj71139-bib-0050] Takahashi, Y. , Zhang, J.B. , Hsu, P.K. , Ceciliato, P.H.O. , Zhang, L. , Dubeaux, G. et al. (2020) MAP3Kinase‐dependent SnRK2‐kinase activation is required for abscisic acid signal transduction and rapid osmotic stress response. Nature Communications, 11, 1. Available from: 10.1038/s41467-019-13875-y PMC6940395 31896774

[tpj71139-bib-0051] Tian, S.J. , Mao, X.G. , Zhang, H.Y. , Chen, S.S. , Zhai, C.C. , Yang, S.M. et al. (2013) Cloning and characterization of *TaSnRK2*.3, a novel SnRK2 gene in common wheat. Journal of Experimental Botany, 64(7), 2063–2080. Available from: 10.1093/jxb/ert072 PMC3638835 23630328

[tpj71139-bib-0052] Ullah, A. , Zhao, C.F. , Zhang, M.X. , Sun, C.J. , Liu, X.X. , Hu, J.L. et al. (2023) Nitrogen enhances the effect of pre‐drought priming against post‐anthesis drought stress by regulating starch and protein formation in wheat. Physiologia Plantarum, 175, 2. Available from: 10.1111/ppl.13907 37039612

[tpj71139-bib-0053] Wang, G.M. , Xu, Y. , Guan, S.L. , Zhang, J.Y. , Jia, Z.H. , Hu, L.J. et al. (2024) Comprehensive genomic analysis of CiPawPYL‐PP2C‐SnRK family genes in pecan (Carya illinoinensis) and functional characterization of CiPawSnRK2.1 under salt stress responses. International Journal of Biological Macromolecules, 279, 3. Available from: 10.1016/j.ijbiomac.2024.135366 39244129

[tpj71139-bib-0054] Wang, M. , Zhang, P.L. , Liu, Q. , Li, G.J. , Di, D.W. , Xia, G.M. et al. (2020) TaANR1‐TaBG1 and TaWabi5‐TaNRT2s/NARs link ABA metabolism and nitrate Acquisition in Wheat Roots. Plant Physiology, 182(3), 1440–1453. Available from: 10.1104/pp.19.01482 PMC7054875 31937682

[tpj71139-bib-0055] Wang, W. , Hu, B. , Yuan, D.Y. , Liu, Y.Q. , Che, R.H. , Hu, Y.C. et al. (2018) Expression of the nitrate transporter gene *OsNRT1*.*1A*/*OsNPF6*.*3* confers high yield and early maturation in Rice. Plant Cell, 30(3), 638–651. Available from: 10.1105/tpc.17.00809 PMC5894839 29475937

[tpj71139-bib-0056] Wang, X.N. , Chai, X.F. , Gao, B.B. , Deng, C. , Günther, C.S. , Wu, T. et al. (2023) Multi‐omics analysis reveals the mechanism of bHLH130 responding to low‐nitrogen stress of apple rootstock. Plant Physiology, 191(2), 1305–1323. Available from: 10.1093/plphys/kiac519 PMC9922409 36417197

[tpj71139-bib-0057] Wang, X.N. , Zhou, Y. , Chai, X.F. , Foster, T.M. , Deng, C.H. , Wu, T. et al. (2024) miR164‐MhNAC1 regulates apple root nitrogen uptake under low nitrogen stress. The New Phytologist, 242(3), 1218–1237. Available from: 10.1111/nph.19663 38481030

[tpj71139-bib-0058] Wang, Y. , Wen, X.L. , Lv, Z.C. , Chen, H.X. , Liang, D. , Xia, H. et al. (2025) Genome‐wide identification of SnRK gene family and functional characterization of AcSnRK2.4 in response to heat stress in kiwifruit. Environmental and Experimental Botany, 233. Available from: 10.1016/j.envexpbot.2025.106146

[tpj71139-bib-0059] Wu, X.Y. , Liu, T. , Zhang, Y.J. , Duan, F.Y. , Neuhäuser, B. , Ludewig, U. et al. (2019) Ammonium and nitrate regulate NH_4_ ^+^ uptake activity of Arabidopsis ammonium transporter AtAMT1;3 via phosphorylation at multiple C‐terminal sites. Journal of Experimental Botany, 70(18), 4919–4929. Available from: 10.1093/jxb/erz230 PMC6760267 31087098

[tpj71139-bib-0060] Wu, Y. , Yang, W. , Wei, J. , Yoon, H. & An, G. (2017) Transcription factor OsDOF18 controls ammonium uptake by inducing ammonium transporters in Rice roots. Molecules and Cells, 40(3), 178–185. Available from: 10.14348/molcells.2017.2261 PMC5386955 28292004

[tpj71139-bib-0061] Xiang, D.J. , Man, L.L. , Cao, S. , Liu, P. , Li, Z.G. & Wang, X.D. (2020) Heterologous expression of an *Agropyron cristatum* SnRK2 protein kinase gene (*AcSnRK2.11*) increases freezing tolerance in transgenic yeast and tobacco. 3 Biotech, 10, 5. Available from: 10.1007/s13205-020-02203-7 PMC7181469 32351867

[tpj71139-bib-0062] Xu, N. , Yu, B. , Chen, R.R. , Li, S.T. , Zhang, G.C. & Huang, J.L. (2020) *OsNAR2.2* plays a vital role in the root growth and development by promoting nitrate uptake and signaling in rice. Plant Physiology and Biochemistry: PPB, 149, 159–169. Available from: 10.1016/j.plaphy.2020.02.004 32070909

[tpj71139-bib-0063] Ye, Y. , Jia, X. , Xue, M.Y. , Gao, Y.C. , Yue, H. , Ma, F.W. et al. (2022) *MpSnRK2.10* confers salt stress tolerance in apple via the ABA signaling pathway. Scientia Horticulturae, 298. Available from: 10.1016/j.scienta.2022.110998

[tpj71139-bib-0064] Zhang, X. , Cui, Y.N. , Yu, M. , Su, B.D. , Gong, W. , Baluska, F. et al. (2019) Phosphorylation‐mediated dynamics of nitrate Transceptor NRT1.1 regulate auxin flux and nitrate signaling in lateral root growth. Plant Physiology, 181(2), 480–498. Available from: 10.1104/pp.19.00346 PMC6776865 31431511

[tpj71139-bib-0065] Zhang, X.L. , Gong, X.Q. , Su, X.J. , Yu, H.X. , Cheng, S.Y. , Huang, J.W. et al. (2023) The ubiquitin‐binding protein MdRAD23D1 mediates drought response by regulating degradation of the proline‐rich protein MdPRP6 in apple (*Malus domestica*). Plant Biotechnology Journal, 21(8), 1560–1576. Available from: 10.1111/pbi.14057 PMC10363924 37140026

[tpj71139-bib-0066] Zhang, Z.J. , Yang, C. , Xi, J. , Wang, Y.T. , Guo, J. , Liu, Q.W. et al. (2024) The MdHSC70‐MdWRKY75 module mediates basal apple thermotolerance by regulating the expression of heat shock factor genes. The Plant Cell, 36(9), 3631–3653. Available from: 10.1093/plcell/koae171 PMC11371167 38865439

[tpj71139-bib-0067] Zhao, Z.Y. , Zheng, W. , Ma, Y.T. , Wang, X.L. , Li, Z.Y. , Zhai, B.N. et al. (2020) Responses of soil water, nitrate and yield of apple orchard to integrated soil management in loess plateau, China. Agricultural Water Management, 240. Available from: 10.1016/j.agwat.2020.106325

[tpj71139-bib-0068] Zhong, R.L. , Wang, Y.X. , Gai, R.N. , Xi, D.D. , Mao, C.J. & Ming, F. (2020) Rice SnRK protein kinase *OsSAPK8* acts as a positive regulator in abiotic stress responses. Plant Science, 292. Available from: 10.1016/j.plantsci.2019.110373 32005379

[tpj71139-bib-0069] Zhuo, M. , Sakuraba, Y. & Yanagisawa, S. (2024) Dof1.7 and NIGT1 transcription factors mediate multilayered transcriptional regulation for different expression patterns of NITRATE TRANSPORTER2 genes under nitrogen deficiency stress. The New Phytologist, 242(5), 2132–2147. Available from: 10.1111/nph.19695 38523242

